# Positive Parenting and Early Childhood Cognition: A Systematic Review and Meta-Analysis of Randomized Controlled Trials

**DOI:** 10.1007/s10567-022-00423-2

**Published:** 2023-02-02

**Authors:** Heather Prime, Krysta Andrews, Alexandra Markwell, Andrea Gonzalez, Magdalena Janus, Andrea C. Tricco, Teresa Bennett, Leslie Atkinson

**Affiliations:** 1 Department of Psychology, York University, Toronto, Canada; 2 LaMarsh Centre for Child & Youth Research, York University, Toronto, Canada; 3 Offord Centre for Child Studies, Department of Psychiatry and Behavioural Neurosciences, McMaster University, Hamilton, Canada; 4 Knowledge Translation Program, Li Ka Shing Knowledge Institute, St. Michaels Hospital, Unity Health Toronto, Toronto, Canada; 5 Epidemiology Division and Institute of Health Policy, Management, and Evaluation, Dalla Lana School of Public Health, University of Toronto, Toronto, Canada; 6 Queen’s Collaboration for Health Care Quality Joanna Briggs Institute Centre of Excellence, Queen’s University, Kingston, Canada; 7 Department of Psychology, Toronto Metropolitan University, Toronto, Canada

**Keywords:** Positive parenting, Early childhood cognition, Randomized controlled trials, Systematic review, Meta-analysis

## Abstract

This review examines the effectiveness of positive parenting interventions aimed at improving sensitivity, responsiveness, and/or non-harsh discipline on children’s early cognitive skills, in four meta-analyses addressing general mental abilities, language, executive functioning, and pre-academics. The objectives are to assess the magnitude of intervention effectiveness and identify moderators of effectiveness. We include randomized controlled trials of interventions targeting positive parenting to improve cognition in children < 6 years. Studies that include children with neurodevelopmental and/or hearing disorders were excluded. MEDLINE, PsycINFO, ERIC, and ProQuest Dissertations & Theses (October 2021) and citation chaining identified relevant records. Five reviewers completed screening/assessments, extraction, and risk of bias. Pooled analysis in Comprehensive Meta-Analysis (Version 3) used random effects modeling, with moderation via *Q-*statistics and meta-regression. Positive parenting interventions led to significant improvements in mental abilities (*g* = 0.46, *N* = 5746; *k* = 33) and language (*g* = 0.25, *N* = 6428; *k* = 30). Effect sizes were smaller and nonsignificant for executive functioning (*g* = 0.07, *N* = 3628; *k* = 14) and pre-academics (*g* = 0.16, *N* = 2365; *k* = 7). Robust moderators emerged for language and cognition. For cognition, studies with higher risk of bias scores yielded larger intervention effects. For language, studies with younger children had larger effect sizes. Studies mitigated selection and detection bias, though greater transparency of reporting is needed. Interventions that promote parental sensitivity, responsiveness, and non-harsh discipline improve early mental abilities and language. Studies examining executive functioning and pre-academics are needed to examine moderators of intervention effectiveness.

*Trial registration* Systematic review PROSPERO registration. CRD42020222143

**Supplementary Information:**

The online version contains supplementary material available at 10.1007/s10567-022-00423-2.

Children’s early cognitive skills lay the foundation for lifelong learning and well-being. Individual differences in mental abilities, language, executive control, and early literacy are linked to preschool and school-age learning, as well as adulthood achievement, educational, and occupational outcomes (Ahmed et al., 2019; Johnson et al., 2010; Scarborough et al., 2009; Wade et al., 2018). Furthermore, early cognitive systems underlie general risk for psychopathology (Michelini et al., 2021; Morris & Cuthbert, 2012). Thus, early cognitive skills represent transdiagnostic risk markers for a host of short- and long-term clinical, school, and family outcomes. Identifying modifiable contributors to early cognition is essential to policy and programming designed to reduce early disparities in development.

Children’s developing competencies are, in part, constructed within cooperative social exchanges (Carpendale & Lewis, 2004). The mutually responsive stance between a child and their parent(s) is built within a positive parent–child relationship. That is, a parent who is sensitive to their child’s subtle and overt cues promotes an eager, willing stance in the child, who then reciprocates the exchange within and across interactions over time (Kochanska et al., 2015). This dynamic creates fertile grounds for the parent learning about the needs of the child and tailoring their input accordingly, while also motivating the child to engage and commit to learning in their social environments—both within the parent–child relationship and beyond. The mutually responsive stance is also important in the disciplinary context; for instance, a strong relational foundation between a parent and a child may lead a child to accept parents’ bids for power and control, rather than interpreting such acts as hostile or threatening (and thus responding in an oppositional manner; Kochanska et al., 2009).

The current paper examines a collection of unique though overlapping parenting behaviours that are considered beneficial to young children including sensitivity, responsiveness, and non-harsh discipline (i.e., positive behavioural management). There is not a unified definition of these parenting behaviours, though they are commonly targeted together (Landry et al., 2008), and they have previously been referred to as *positive parenting* (Juffer et al., 2008; Madigan et al., 2019; Sanders et al., 2014). As such, we will refer to this collection of parenting behaviours as positive parenting, hereafter. Though this general term has the advantage of referring to varied yet related parenting behaviours, it has the disadvantage of lacking specificity.

Positive parenting takes different forms based on the currently activated relationship between parent and child (Grusec, 2011). Traditionally conceptualized within an attachment framework, positive parenting behaviours can be characterized by sensitivity, warmth, acceptance, and nurturance (Ainsworth, 1979), as well as consideration of infants’ intentions, thoughts, and emotions (i.e., mind-mindedness; Laranjo et al., 2008). Relatedly, positive parenting can be understood within a sociocultural framework, wherein parents respond promptly and contingently to infants’ exploratory and communicative actions, serving to expand their individual learning through an interpersonal exchange (Bernier et al., 2010; Tamis-Lemonda et al., 2014). Thus, both cognitively and affectively responsive behaviours characterize positive parenting in infancy and throughout early childhood (Landry et al., 2008). As infants enter their second year, their cognitive, linguistic, and physical development leads to aggression and active resistance to parental control (Alink et al., 2006; Côté et al., 2006). As a result, what is considered positive parenting expands to include non-harsh discipline, appropriate limit-setting, and monitoring of child behaviour, within the realm of social learning and operant conditioning frameworks. Thus, positive parenting behaviours vary as a function of context (e.g., cooperative exchanges such as play; hierarchical exchanges such as teaching, protecting, or limit-setting) and/or child needs based on age, neurodevelopmental and/or temperamental characteristics (Grusec, 2011). Cutting across these positive parenting behaviours is a motivation and capacity to attend to the internal states of the child, reasonably respect their needs for autonomy, and cultivate a warm relationship.

Evidence for the predictive power of positive parenting in relation to children’s early cognition comes primarily from naturalistic, longitudinal study designs. For instance, Browne et al. (2018) demonstrated that the relationship between socioeconomic, neighbourhood, and household risk (child age 2 months) and children’s pre-academic skills, vocabulary, executive function, and theory of mind (4.5 years), operated, in part, through parental responsiveness (18 months), after accounting for family material investments. Furthermore, executive functioning has been longitudinally associated with early positive parenting, such as sensitivity, positive regard, and stimulation (Towe-Goodman et al., 2014), and serves as a link between these early parenting behaviours and children’s later behavioural problems (Sulik et al., 2015). Importantly, children whose mothers show more responsive behaviours in infancy *and* toddlerhood demonstrate enhanced cognitive and language skills, as compared to those whose mothers show more responsive behaviours in only infancy or early childhood (Landry et al., 2001). Robust support for associations between positive parenting and early cognition comes from reviews and meta-analyses, for instance in children’s language and mental abilities (Madigan et al., 2019; Neel et al., 2018), academic achievement (Pinquart, 2016), and executive functioning (Valcan et al., 2017).

Although there is robust correlational evidence that positive parenting behaviours are linked to early childhood cognition, study designs that can establish causal processes are needed. Behavioural genetic studies demonstrate that early cognitive development is, in part, genetically mediated (Friedman et al., 2008; Hayiou-Thomas et al., 2006; Polderman et al., 2015; van Bergen et al., 2018). Moreover, putative environmental effects are confounded by genetics by way of evocative gene-environment correlations (e.g., parental warmth is significantly influenced by children’s genetic propensities; Klahr & Burt, 2014) and passive gene-environment correlations (e.g., reading ability is primarily transmitted from parent to child through genes rather than the home-literacy environment; van Bergen et al., 2017). As such, observed associations between parenting and early development are, in part, confounded by genetic factors. Furthermore, even in well-controlled observational designs, associations between environmental and child outcomes are at risk of confounding by unmeasured environmental factors (e.g., family functioning; see Daniel et al., 2018 for an example). Given these confounds, researchers and consumers of research may make erroneous conclusions that the home environment and children’s outcomes are causally linked without sufficient evidence (Haber et al., 2018, 2021; Hart et al., 2021).

Randomized controlled trials (RCTs) confer a high probability for establishing causal mechanisms, with differences in outcomes across intervention and control groups attributed to the effects of the intervention (Cook et al., 2002). A growing number of RCTs have examined positive parenting interventions in relation to early cognitive development, including language (Bagner et al., 2016; Guttentag et al., 2014), mental abilities (Dubois-Comtois et al., 2017; Roggman et al., 2009), executive functioning (Cassidy et al., 2017; Lunkenheimer et al., 2008), and literacy and numeracy skills (Landry et al., 2021; Sheridan et al., 2011). Studies yield variable estimates when it comes to the direction and magnitude of intervention effects, and there are considerable differences in study design in terms of participant, intervention, and outcome characteristics (Bernard et al., 2017; Boivin et al., 2017; Cassidy et al., 2017; Colditz et al., 2019; Hutchings et al., 2017; Pontoppidan et al., 2020; Tachibana et al., 2012). Thus, although there is growing support for the effectiveness of positive parenting interventions in enhancing children’s early cognition, several questions remain, which are best addressed with a systematic review and meta-analysis.

The current study is a systematic review and meta-analysis designed to illustrate the state of knowledge regarding the effectiveness of positive parenting interventions, based on RCT designs, for enhancing children’s early cognition. In a recent systematic review and meta-analysis, Jeong et al. (2021) examined 102 unique RCTs of a range of parenting interventions delivered during the first three years of life. All studies had an evaluation of an early childhood development outcome, among which included cognitive and/or language development. Parenting interventions that included content to promote responsive parenting behaviours (i.e., prompt, consistent, contingent, and developmentally appropriate to the child’s cues, signals, and needs) were more effective at enhancing child cognitive development as compared to parenting interventions without a focus on promoting responsive parent–child interactions. Thus, content that focuses on positive parent–child interactions may be necessary to optimize the effectiveness of parenting interventions in the first 3 years of life. However, it is unclear whether positive parenting interventions, in isolation, are effective in enhancing children’s early cognitive development, as several studies in the review had additional intervention targets (e.g., the provision of early play and learning materials, caregiver awareness of developmental milestones). The current study aim is to shed light on the utility of single-focused interventions (i.e., is positive parenting content sufficient?), while also informing developmental theory on child cognition and the parenting environment.

Furthermore, the current study extends Jeong et al. (2021) review by including children up to and including six years of age. This has several advantages, including greater sensitivity to detect potential age effects, the inclusion of intervention strategies designed to address the needs of preschool children or a wider range of children (e.g., behavioural guidance), and the assessment of skills that develop later in early childhood such as executive functioning and pre-academics.

## Potential Moderators

### Child Characteristics

Child-specific characteristics, such as age, sex, and early risk factors, may impact the effectiveness of positive parenting interventions. Whereas there is some meta-analytic evidence for greater effects of positive parenting interventions for younger children as compared with older children when examining socio-emotional and behavioural outcomes (Gardner et al., 2010; Sanders et al., 2014), other meta-analyses do not support this claim (Gardner et al., 2019b; Van Aar et al., 2017). Findings in relation to positive parenting interventions and cognitive development are similarly mixed (Baudry et al., 2017; Jeong et al., 2021). Timing effects may depend on the age range of the samples included and/or the developmental outcome examined (Maughan & Barker, 2019).

There is evidence for differences in male versus female children in cognitive development, positive parenting, and the relations between positive parenting and children's outcomes (Barnett & Scaramella, 2013; Else-Quest et al., 2006; Leaper & Smith, 2004). Previous reviews are mixed with respect to whether child sex moderates the effectiveness of positive parenting interventions, with evidence for stronger intervention effects in male children (Gardner et al., 2017), and no differences between male and female children (Nowak & Heinrichs, 2008).

Finally, early infant/childhood risk factors such as peri-/postnatal problems (e.g., low birth weight, prematurity) and/or socio-emotional/behavioural problems may impact intervention effectiveness. Stronger effect sizes have been observed in parenting interventions targeted towards higher risk children in relation to behavioural outcomes, such as those with developmental needs and those with higher distress at baseline (Gardner et al., 2017; Sanders et al., 2014). There is alternative evidence that children with peri-/postnatal risk may not make the same cognitive gains as those without such risks following parent intervention (Landry et al., 2008; Vanderveen et al., 2009). However, this question has not be examined meta-analytically in relation to cognitive outcomes.

### Contextual Characteristics

Social disadvantage is consistently related to children’s early cognitive development, partially explained by family factors such as parenting; this has been robustly demonstrated for both socioeconomic status (Borairi et al., 2021; Letourneau et al., 2013) and maternal depression (Ahun & Côté, 2019; Liu et al., 2017). It is important to understand whether parenting interventions focused on promoting positive parenting behaviours, without additional program components, are effective for families experiencing multiple stressors. The effectiveness of the Incredible Years parenting program, designed to support children’s behavioural functioning, is not reduced for disadvantaged families (Gardner et al., 2019a, 2019b), and indeed confers greater effects for some higher risk families such as those with depressed parents (Gardner et al., 2017). However, such findings cannot be directly applied to children’s cognitive development, where there is contradictory evidence for the effectiveness of positive parenting programs with high-risk families (Baudry et al., 2017; Rayce et al., 2020).

### Intervention Characteristics

Both parental affective- and cognitive responsiveness have been linked to gains in children’s cognitive development following parent training (Landry et al., 2008). However, distinctions between specific positive parenting behaviours (e.g., warmth and contingent responding, respectively) are evident, in terms of the strength of their prediction of child development (Borairi et al., 2021; Madigan et al., 2019; Neel et al., 2018). Thus, we will examine whether there is an association between parenting behaviour targeted and intervention effect size. Intervention format is relevant, too; interventions that have fewer sessions and those that are shorter in duration have been shown to be more effective for inciting change in parent and child outcomes (Bakermans-Kranenburg et al., 2003; Baudry et al., 2017; Harris et al., 2020; Jeong et al., 2021). Finally, given that fathers are frequently overlooked in research on child psychopathology (Parent et al., 2017), one goal of the current study is to systematically examine how frequently fathers are included in positive parenting interventions designed to enhance children’s cognitive development, and to examine whether inclusion influences differences in intervention effects.

### Methodological Characteristics

Several methodological characteristics influence between-study variability in effect sizes, including year of publication, risk of bias/methodological quality, and publication status (Baudry et al., 2017; Nowak & Heinrichs, 2008; Pinquart, 2016, 2017). In addition, the method of child outcome assessment (e.g., parent-report vs. direct assessment) can influence the strength of effects, as seen in both in individual studies and meta-analyses (Andrews et al., 2021; Landry et al., 2017; Madigan et al., 2013; Nowak & Heinrichs, 2008).

## Current Study

The current study is a systematic review and meta-analysis that includes primary studies of positive parenting interventions in the infancy and early childhood period. All primary studies in the review include RCT designs and an evaluation of one or more of mental abilities, language, executive functioning and/or pre-academics. The goal of the review is to obtain a pooled estimate of the magnitude of the effect of positive parenting programs for promoting positive gains in early child cognition, and to identify moderating factors associated with intervention effect sizes.

All included studies evaluate single-focused interventions in that they have a primary focus on positive parenting with only minimal, if any, additional intervention components. Notably, interventions that target positive parenting behaviour draw on several theoretical models including attachment, social learning, sociocultural, and biobehavioural frameworks (Prime et al., 2020). The current review uses a comprehensive definition of positive parenting that includes emotional responsiveness and sensitivity (e.g., responding to distress, affection/warmth), cognitive responsiveness (e.g., maintaining children’s focus of attention, responding to infants’ exploratory and communicative actions), and positive behavioural guidance/management. We do not include parenting interventions that are specifically literacy-based or focused solely on enhancing parental language input.

We use early cognitive skills as a broad umbrella term for four categories of foundational early learning: mental abilities, language, executive functioning, and pre-academics. Mental abilities are typically represented by developmental/intelligence quotients, and/or cognition scores, based on assessments of a variety of skills such as visuo-spatial, motor, quantitative, and verbal/non-verbal reasoning skills. Language refers to children’s verbal abilities, including communication, receptive (i.e., understanding) and expressive (i.e., production) vocabulary, and speech production. Executive functioning refers to children’s developing abilities for inhibition, cognitive flexibility/set-shifting, effortful control, and working memory. Finally, pre-academics represents acquired knowledge in the domains of achievement, early literacy (e.g., print knowledge), numeracy (e.g., counting), and related skills (e.g., shapes and colours). There is significant interdependence among this subset of early skills, in early childhood and into the elementary school years (Fitzpatrick et al., 2014; Spiegel et al., 2021), making it useful to examine in a unified project (Prime et al., 2020; Rodrigues et al., 2021). At the same time, there are differential effects of early psychosocial interventions across early cognitive skills, for instance, with some evidence of stronger benefits to broad developmental, language, and pre-academic skills, as compared to executive functioning (Bick et al., 2018; Landry et al., 2017; McDermott et al., 2012). The current study examines these four domains of early child cognition, independently, to assess whether there are differential patterns of effectiveness of positive parenting interventions and/or moderating factors.

First, we systematically review studies to draw themes in study, participant, and intervention characteristics, while also providing a critical assessment of the strengths and weaknesses of the extant literature through a risk of bias assessment. Second, we examine outcomes independently in four separate meta-analyses, with accompanying outcome-specific moderator analyses. Several substantive and methodological moderators are examined as potential explanations for between-study heterogeneity of effect sizes, including child characteristics (age, sex, early risk factors), contextual characteristics (income, education, parental age, parental mental health), intervention characteristics (parenting target, intensity, duration, father involvement), and methodological characteristics (publication year, publication status, sample size, risk of bias, outcome assessment approach).

## Methods

### Study Inclusion Criteria

Eligibility criteria are based on population, intervention, comparators, outcomes, and study design (PICOS) characteristics and include: (i) a study population of children less than or equal to 6 years of age; (ii) an intervention with a primary focus on improving positive parenting behaviours, as defined as one or more of the following: emotional responsiveness (e.g., sensitivity, warmth, contingent responding, acceptance), cognitive responsiveness (e.g., rich verbal input, following attention, verbal scaffolding), and/or positive behavioural guidance (e.g., incentives, limit-setting and non-harsh discipline, reinforcement/praise)^1^; (iii) a comparison group utilizing passive control, treatment-as-usual, and/or waitlist controls (but not active controls that receive a comparable standard treatment.); (iv) an assessment of children’s cognitive development at less than or equal to six years of age, as defined by mental abilities (e.g., cognition, developmental/intelligence quotient, reasoning/problem-solving, performance), language (e.g., verbal abilities, communication, receptive/expressive vocabulary, speech production), executive functioning (e.g., inhibition, cognitive flexibility/set-shifting, effortful control, working memory), and/or pre-academics (e.g., print knowledge, school readiness, achievement); and (v) a randomized controlled trial study design. Only articles written in English were included. Published and unpublished records were considered. Exclusion criteria included studies with samples of parents/children with intellectual disabilities, deafness/hearing loss, blindness, and brain injuries.

### Information Source and Search Strategy

The search strategy was developed and executed by the first author, in consultation with an experienced librarian, experts in knowledge synthesis research methods, and the research team (remaining authors). First, a database search of MEDLINE (Ovid), PsycINFO (Ovid), ERIC, and ProQuest Dissertations & Theses Global was executed in September 2020 (from inception to date of the search) and updated in October 2021. The search strategy went through multiple rounds of pilot testing (Hayman, 2015). The final strategy included a combination of subject terms/headings and keywords tailored to individual databases indexing: parenting, intervention, early childhood, and cognition (see Supplemental File 1 for the entire database search strategy). Eligible studies identified at the full-text phase were put through backward (reference lists) and forward (cited by lists) searching procedures to identify additional records for screening (Boland et al., 2017).

### Study Records

#### Data Management

Records identified in the search strategy were imported into Covidence (Covidence, 2017), and duplicates removed. Abstract/title screen, full-text assessment, data extraction of study characteristics, and risk of bias assessment were conducted in Covidence, and extraction of effect sizes was executed in Microsoft Excel.

#### Selection Process

Standardized manuals were developed by the first author and piloted by the research team for abstract screening (50 records) and full-text assessments (40 full-texts). Interrater agreement was established prior to independently screening/assessing records and full-texts (Percent agreement ≥ 0.80; Kappa ≥ 0.60). The abstract screening was completed by four members of the research team, with each abstract screened by two independent reviewers, and discrepancies resolved by the first author. Full-text assessments were similarly executed by two members of the research team, with discrepancies resolved by the first author.

#### Data Collection Process

Data extraction forms were developed by the first author. The data extraction forms went through piloting on five articles, with multiple iterations, to enhance clarity and inter-rater agreement. Teams of two completed extraction of either study characteristics, effect size extraction, or risk of bias assessments. Discrepancies were resolved by a third senior reviewer or through discussion and consensus (for effect size extraction). Subsequently, all data extraction was reviewed by the first author. First and senior authors of primary studies were contacted for three primary reasons: (i) to request full-text articles that were not readily available online or through library services; (ii) to request data required to compute an effect size (including means/standard deviations and/or pre-post correlations, as described below in Meta-Analysis); and (iii) to request missing data related to study characteristics.

### Data Items

Data items were selected based on the Cochrane Handbook, study objectives, and norms of the child development and parenting literature. *Participant characteristics* included child age (in months) at baseline and at the time of outcome assessment, and % of male children. Most risk indicators were dichotomized as present or absent in a sample based on whether ≥ 30% of the sample was deemed to have the risk present. An exception to this was for parental income and education, which were considered present if ≥ 50% of the sample was deemed to have the risk.^2^ Child-related risks were coded as peri/post-natal risks (e.g., low birth weight, prematurity) and/or social-emotional/behavioural risks (as indicated, for example, by elevated scores on a screener measure). Parental risks included low education (≤ a high school education; or low education as defined by authors) and/or low income (equivalent to ≤ $40,000 USD; or low income as defined by authors). Additional risk factors included whether the sample was made up of adolescent parents and the presence of parental mental health difficulties. We were able to code the mean parental age (in years) continuously. *Intervention characteristics* included the intervention name, intensity (16 + sessions vs. < 16; Bakermans-Kranenburg et al., 2003), duration (12 + months vs. < 12 months; Jeong et al., 2021), % mothers participating, father involvement (1 = any indication of involvement; 0 = no indication of father involvement), who the intervention was delivered by, intervention setting (e.g., home, community, hospital), and the nature of the comparison group. The specific target of positive parenting was coded as affective-emotional responsiveness, cognitive responsiveness, behavioural guidance, or mixed (1 + of the preceding targets). *Methodological characteristics* included year of publication, type of record (journal article, dissertation, book chapter, report)*,* country of origin, the sample size (analytic sample), and the outcome measurement approach (i.e., standardized assessment, parent-reported, or behavioural observation).

### Outcomes

The four primary outcomes for the systematic review and meta-analysis were:

(i) Mental abilities (e.g., cognition, developmental/intelligence quotient, reasoning/problem-solving, performance);
(ii) Language (e.g., verbal abilities, communication, receptive/expressive vocabulary, speech production);
(iii) Executive functioning (e.g., inhibition, cognitive flexibility/set-shifting, effortful control, working memory) and/or;
(iv) Pre-academics (e.g., print knowledge, school readiness, achievement).

#### Moderators

Moderators that were examined to explain between-study variation in effect sizes include outcome, child, contextual, intervention, and methodological characteristics, as listed in Table 6.

### Risk of Bias in Individual Studies

A risk of bias assessment was conducted for included studies using the modified Cochrane risk of bias tool for randomized trials, developed by the CLARITY Group at McMaster University (CLARITY Group, 2021). The Clarity tool was selected as it provides variability in the classification system based on available information (e.g., probably yes and/or probably no, in addition to definitely yes/definitely no options). Furthermore, it provides specific instructions for addressing unclearly reported masking status in randomized controlled trials (Akl et al., 2012). These modifications allow for a nuanced assessment of risk of bias when a literature has variability in the quality of reporting, as was expected in the current review.

Studies were evaluated based on the following criteria: adequacy of random sequence generation, adequacy of allocation concealment, masking to group allocation (data collectors, participants, interventionists, data analysts), frequency of missing outcome data, selective outcome reporting, and other biases (e.g., baseline differences). Additional questions were added to the assessment after consultation with the tool developer including the use of a power analysis, sample power, and trial registration. For each criterion, studies were rated as either: (1) definitely high-risk of bias; (2) probably/mostly high-risk of bias; (3) probably/mostly low risk of bias, or (4) definitely low risk of bias. Following piloting and adjustment of the assessment with 11 articles, each study was assessed by two independent coders and disagreements resolved by the second author. For multiple studies pulling from the same sample, ratings were compared for each criterion and where discrepancies emerged (*k* = 6), the higher rating was selected. Finally, total scores were generated for each study by assigning a value of 1 for “Definitely Yes” or “Probably Yes”, and 0 for “Definitely No, Probably No or Not Reported”, and then summing across all risk of bias items. Scores could range from 0 to 12 with higher scores indicating lower risk of bias.

### Data Synthesis

#### Description of Studies

Descriptive tables are presented with individual-study data, including participant, intervention, and methodological characteristics, respectively. Frequencies (and percentages) and means/standard deviations are used to summarize descriptive information across studies, when applicable. Risk of bias assessment is summarized with frequencies and percentages, and a visual depiction of individual-level data by study is presented.

#### Quantitative Analyses

All studies with sufficient data to compute an effect size were included in the meta-analysis. Authors were contacted to request data when sufficient data were not readily available. Studies with insufficient data to compute an effect size were excluded from the meta-analysis (though retained in the qualitative synthesis); however, sensitivity analyses were used to examine the impact of inclusion/exclusion of these studies on the pooled effect sizes, with an effect of 0.0 substituted for missing data. Comprehensive Meta-Analysis Version 3 software (Borenstein et al., 2013) was used to calculate individual effect sizes as Hedge’s *g* (Hedges, 1981) and, subsequently, to pool effects across studies. We included the pre- and post means and standard deviations of intervention and control groups, respectively, and correlations between pre- and post-test scores, to obtain standardized difference scores (Borenstein et al., 2021). Pre-post score correlations were requested from authors when not reported. When not available, pre-post score correlations were imputed at 0.7 (with sensitivity analyses at 0.5; Rosenthal, 1991). When pre/post means and standard deviations were not available, other methods were used to obtain an effect size (e.g., post-test score comparison, correlations between group and post-test outcome, sample size and *p*-value, etc.). Four meta-analyses were conducted using random effects modeling and independent samples: one for each of mental abilities, language, executive functioning, and pre-academics, respectively, including outcome-specific moderation analyses. These four meta-analyses were not independent (i.e., samples between meta-analyses were overlapping).

Publication bias was examined for each meta-analysis using the trim-and-fill approach to assess degree of possible test bias, if any (Duval & Tweedie, 2000). In the event of evidence of publication bias, Rosenthal's (1986) *Fail-safe N* was examined, as an additional indicator of potential publication bias, to estimate the number of unpublished studies with null results that would deem the effect size nonsignificant. Publication bias could not be examined via moderation analyses due to small cell sizes within each meta-analysis (i.e., < 5 unpublished studies in all relevant cells).

#### Effect Size Selection

Several studies drew from overlapping samples. We screened for overlapping samples based on first/senior authorship and/or intervention names (e.g., Incredible Years, Family Check-Up), and confirmed overlap by comparing study characteristics. In addition, several studies had multiple outcomes of interest either across outcomes (e.g., language and executive functioning), within outcomes over time (language at post-intervention and follow-up), or both. To ensure each sample was only represented in each meta-analysis once, the following steps were taken prior to data analysis:

(i) The broadest level of measurement was typically retained (e.g., an executive functioning composite of three subscales was selected over any single executive functioning subscale). The exception was when the broadest level of measurement included two domains of cognitive development (e.g., mental abilities and language), in which case we retained outcomes across domains, as these outcomes were analyzed in independent meta-analyses.
(ii) The measurement approach that most robustly protected against risk of bias was retained (standardized assessment > observations > parent-report). Outcomes that used the same measurement approach (e.g., both parent-report) were combined. To combine two or more effect sizes into a single effect size, an estimate of the correlations among outcomes is required (Borenstein et al., 2021). As these were typically not available, a plausible range of correlations was used, wherein we imputed a correlation between variables of 0.2 (and sensitivity analyses were conducted with an assumed correlation of 0.7).
(iii) We selected the earliest assessment point (most often the post-intervention assessment but sometimes only a follow-up was available). Sensitivity analyses compared this selection to one where we selected the latest assessment point; that is, we used the effect size from the follow-up instead of post-intervention.

#### Moderation Analyses

Heterogeneity of effect sizes (or, dispersion) was assessed using the *Q* statistic, and true dispersion was assessed based on the *I*^2^ statistic. In the presence of significant heterogeneity (i.e., significant *Q* statistic), moderation analyses were conducted (see Table 6 for a list of all moderators). For categorical moderators, moderation was examined using subgroup analyses and significance was assessed via *Q*-statistics. Planned comparisons with two cells were not conducted if one cell included fewer than five samples. Where planned comparisons involved more than two cells, cells with fewer than five samples were excluded from the analysis. Independent meta-regressions were conducted for each continuous moderator, separately (Borenstein et al., 2017, 2021). Subsequently, for each meta-analysis, all significant categorical and continuous moderators were simultaneously entered into a meta-regression to examine the unique variance of each, and their combined linear prediction capacity.

### Transparency and Openness

The systematic review and meta-analysis that follow are guided by the Cochrane Handbook, and reporting complies with the Preferred Reporting Items for Systematic Reviews and Meta-Analyses (PRISMA) statement (Page et al., 2021). The protocol was published (Prime et al. 2021) and it was preregistered with the International Prospective Register of Systematic Reviews (PROSPERO; CRD42020222143). All data and research materials (amendments from the study protocol; screening, full-text assessment, and data extraction manuals) will be made available at APA’s repository on the open science framework (OSF). Data were modeled using Comprehensive Meta-Analysis Version 3; code/syntax is not applicable.

## Results

### Study Selection

**Fig. 1:**
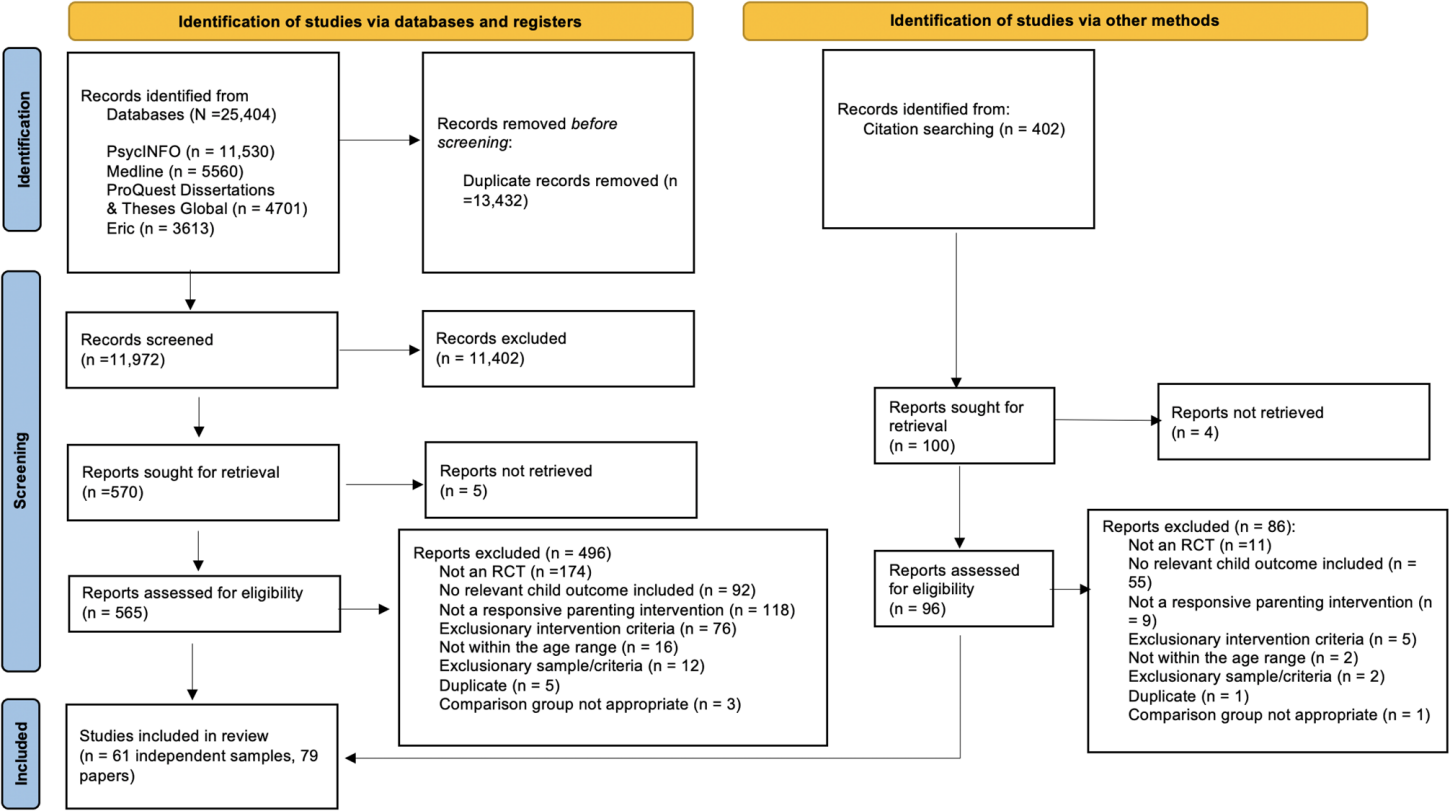
PRISMA 2020 flow diagram for new systematic reviews (Page et al., 2021)

Figure 1 presents the PRISMA flow diagram. After removal of duplicates, there were 11,972 abstract/titles of records screened, with 570 moving to the full-text assessment.^3^ Primary reasons for exclusion from the review were: not an RCT, not a positive parenting intervention, no cognition outcome assessment, and exclusionary intervention criteria (i.e., primary focus on a target other than positive parenting). This resulted in 69 eligible papers. Backward and forward chaining of these papers resulted in screening an additional 402 records, with 96 assessed at the full-text stage, and an additional 10 papers for inclusion (to make 79 total). Papers were examined for overlapping samples, resulting in 61 independent samples across 79 papers.

### Description of Studies

#### Outcome Characteristics

Table 1 provides study-level outcome and participant characteristics. Studies included the following child outcome assessments (not mutually exclusive): mental abilities (*k* = 39, 63.9% of studies), language (*k* = 34, 55.7%), executive functioning (*k* = 14, 23%), and/or pre-academics (*k* = 8, 13.1%). Of these, 35 studies (57.4%) examined one outcome domain only, 20 studies (32.8%) two outcome domains, four studies (6.6%) three outcome domains, and two studies (3.3%) examined all four outcome domains.

**Table 1:** Child and contextual characteristics

Study	Child outcomes				Child age, months (Baseline)^a^	Child age, months (Outcome)^a^	Child, % male	Child, birth risk	Child, socio-emotional risk	Context, low education	Context, low income	Context, parent age	Context, parent mental health
	MA	L	EF	PA									
Sample A (2 papers)^b^		X		X	43.05	61.05	51.20				X	29.44	
Sample B (4 papers)^b^	X	X	X	X	0.68	48.00	53.75	X		X	X	28.20	
Sample D (5 papers)^b^		X	X	X	29.90	36.00	51.00		X	X	X	26.90	
Sample E (2 papers)^b^	X			X	4.00	56.00	54.00	X				27.50	
Sample F (2 papers)^b^		X	X		8.93	13.93	53.70			X	X	–	X
Sample G (3 papers)^b^		X			13.47	15.47	55.00		X	X	X	29.57	
Sample H (2 papers)^b^	X	X			0.00	60.00	52.94	X				30.21	
Sample I (3 papers)^b^	X	X	X		0.00	44.00	50.31	X				34.20	
Sample J (5 papers)^b^	X				0.00	6.00	50.00	X				27.05	
Sample K (2 papers)^b^	X	X			22.00	41.00	43.37			X	X	24.35	
Abikoff et al. (2015)			X		42.84	44.84	73.80		X			–	
Aboud and Akhter (2011)		X			14.16	22.16	46.67			X	X	23.45	
Alvarenga et al. (2020)	X				1.00	12.00	61.36			X	X	27.66	
Bernard et al. (2017)		X			14.80	39.52	44.00					43.20	
Boivin et al. (2013a)	X				33.60	45.60	53.78			X	X	–	
Boivin et al. (2013b)	X				43.80	55.80	54.20			–	X	–	
Boivin et al. (2017)	X		X		34.44	46.44	56.56			X	X	35.00	
Cameron et al. (2021)	X	X			4.00	36.00	38.89	X				30.60	
Cassidy et al. (2017)			X		48.60	50.90	41.80			X	X	29.55	
Cicchetti et al. (2000)	X	X			20.47	36.00	51.20					31.62	X
Colditz et al. (2019)	X	X			0.00	24.00	58.85	X				30.60	
Cooper et al. (2015)	X				0.00	18.00	48.48					28.30	X
Dubois-Comtois et al. (2017)	X				17.76	20.06	51.20			X	X	24.20	
Elizur et al. (2017)			X		48.63	52.08	78.00		X	X	X	34.97	
Feeley et al. (2012)	X	X			1.08	6.00	48.96	X				30.69	X
Feil et al. (2020)		X			4.41	10.41	44.20				X	27.23	X
Fewell and Wheeden (1998)	X				14.56	17.56	51.61			X	X	17.47	
Flierman et al. (2016)	X	X			18.00	24.00	50.00	X				31.00	
Fong et al. (2019)	X				17.40	18.40	52.00			X	X	25.75	
Francis and Baker-Henningham, (2021)				X	48.48	50.32	50.70		X	X	X	31.88	
Guttentag et al. (2014)		X			0.00	30.00	–			X	X	20.49	
Hutchings et al. (2017)	X				21.33	27.33	58.40		X			28.97	
Jensen et al. (2021)	X	X			21.42	36.42	49.72		X	X	X	36.00	
Jin et al. (2007)		X			10.23	16.23	63.00				X	–	
Johnson et al. (2009)	X				0.00	24.26	47.64	X		X	–	29.50	
Jones (2003)			X		34.60	35.52	61.00			–		29.20	X
Kersten-Alvarez et al. (2010)		X			5.50	67.60	60.35	X				30.35	X
Klein and Alony (1993)		X	X		12.07	55.07	50.00			X	X	28.00	
Kyno et al. (2012)	X	X			0.00	36.00	59.64	X				32.25	
Landry et al. (2006)	X				6.20	12.00	48.48	X			X	27.40	
Landry et al. (2017)		X	X	X	52.44	60.44	49.20			X	X	–	
Landry et al. (2021)		X	X	X	50.76	57.76	50.00			X	X	37.17	
Letourneau et al. (2011)	X				5.29	7.99	50.00				X	28.00	X
Madden et al. (1984)^c^	X	X			26.00	50.00	56.00			X	X	31.06	
Madden et al. (1984)^d^	X	X			25.46	49.46	50.00			X	X	28.65	
Madden et al. (1984)^e^	X	X			26.17	50.17	44.52			X	X	29.33	
Madden et al. (1984)^f^	X				25.47	49.47	41.69			X	X	26.49	
Magwaza and Edwards (1991)	X				54.00	56.30	50.00			X	X	35.00	
McManus et al. (2020)	X	X			0.00	6.00	52.63	X			X	–	
Metzl et al. (1980)	X				1.38	6.00	50.00					–	
Milgrom et al. (2013)		X			0.00	6.00	42.21	X				33.75	
Murray et al. (2016)	X				0.00	18.00	52.47			X	X	26.10	X
Newnham et al. (2009)	X	X			0.00	24.00	52.38	X				31.50	
O'Bleness et al. (2015)		X			30.68	33.16	51.61			X	X	27.55	
Pontoppidan et al. (2020)	X				1.50	18.00	54.54					29.33	
Roggman et al. (2009)	X				5.00	36.00	51.00				X	22.84	
Scarr and McCartney (1988)	X	X	X	X	27.00	45.00	50.00			X		27.70	
Semenov et al. (2021)		X	X		49.43	53.43	54.88				X	32.11	
Strayhorn and Weidman (1989)		X			45.00	48.34	44.00		X	X	X	28.40	X
Tachibana et al. (2012)	X				61.56	64.56	48.32			–		33.78	
Walker et al. (2004)	X				0.00	24.00	43.51	X		X	X	23.88	

*MA* mental abilities, *L* language, *EF* executive functioning, *PA* pre-academics

^a^Based on the assessment included in the meta-analysis

^b^Sample A (Clarke et al., 2012; Sheridan et al., 2011); Sample B (Jeong et al., 2019; Obradovic et al., 2016; Yousafzai et al., 2014; Yousafzai et al., 2016); Sample D (Brennan et al., 2013; Chang et al., 2015; Chang et al., 2017; Connell et al., 2019; Lunkenheimer et al., 2008); Sample E (Barrera et al., 1986; Barrera et al., 1991); Sample F (Green et al., 2015; Green et al., 2017); Sample G (Bagner et al., 2016; Garcia et al., 2019; Heymann et al., 2020); Sample H (Kaaresen et al., 2008; Nordhov et al., 2010); Sample I (Koldewijn et al., 2010; van Hus et al., 2013; Verkerk et al., 2012); Sample J (Achenbach et al., 1990, Achenbach et al., 1993; Nurcombe et al., 1984; Rauh et al., 1988; Rauh et al., 1990); Sample K (Slaughter et al., 1979; Slaughter et al., 1983)

^c^1973 Cohort

^d^1974 Cohort

^e^1975 Cohort

^f^1976 Cohort

#### Child and Contextual Characteristics

At baseline, the median age of children was 14.6 months (IQR 1.0–32.1 months), with a range from 0 to 61.6 months old. At the time of outcome assessment, the median child age was 36 months (IQR 18.0–50.1 months), with a range from 6 to 67.6 months old. The mean proportion of male children was 51.8% (range from 38.9 to 78.0%). Several studies had children with early risk factors: eight studies included children with socio-emotional or behavioural problems and 17 studies included children with perinatal or postnatal complications. Around half of studies included parents with less than or equal to high school education (*k* = 31; 50.8%), and many studies included samples of families designated as low income (*k* = 38, 62.3%). Three studies (4.9%) included adolescent parents. Average parent age was 29.3 years (range from 17.5 to 43.2 years). Ten studies (16.4%) included samples of parents reporting mental health difficulties.

#### Intervention Characteristics

As shown in Table 2, there was overlap across studies in the interventions implemented (Mother–Child Home Program, *k* = 5; Play and Learning Strategies, *k* = 4; Mother-Infant Transaction Program, *k* = 4; Mediational Intervention for Sensitizing Caregivers, *k* = 3*;* Incredible Years Parenting Program, *k* = 2; Family Check-Up, *k* = 2). The exact nature of comparison groups is listed in Table 2. In terms of intensity, 24 studies (39.3%) included interventions with 16 + sessions (with the remainder having < 16 sessions). For duration, 20 studies (32.8%) included interventions that lasted 12 months or more (with the remainder being under a year). Interventions targeted emotional responsiveness (*k* = 25, 41%), cognitive responsiveness (*k* = 12, 19.7%), behavioural guidance (*k* = 6, 9.8%) and/or a combination of two or more targets (*k* = 18, 29.5%). Mothers were included in all studies (with a median of 100 % and inter-quartile range of 99.4% to 100%). In contrast, 11 studies (18%) reported father involvement. Interventions were delivered by trained interventionists *(k* = 18, 29.5%; e.g., coaches), clinical health workers (*k* = 13, 21.3%; e.g., physical therapists), mental health professionals (*k* = 10, 16.4%; e.g., psychologists), research staff (*k* = 8, 13.1%), community volunteers (*k* = 4, 6.6%; e.g., peers), educators (*k* = 3, 4.9%; e.g., teachers), or were not classified (*k* = 5, 8.2%).

**Table 2:** Intervention characteristics

Study	Intervention name	16 + Sessions	12 + Months	Parent target	Fathers	Interventionist	Comparison group
Sample A (2 papers)^a^	Getting ready (and head start)		X	M		Educators	Head start only
Sample B (4 papers)^a^	Responsive stimulation intervention (a subset also had enhanced nutrition)	X	X	ER		Community volunteers	Routine health and nutrition services
Sample D (5 papers)^a^	Family check-up	X	X	BG	X	Trained interventionists	Assessment procedures only
Sample E (2 papers)^a^	Parent-infant treatment + developmental intervention (combined)	X	X	ER		Mental health professionals	Assessment schedule only
Sample F (2 papers)^a^	Intervention in the British Autism study of infant siblings-video interaction for promoting positive parenting (iBASIS-VIPP)			ER		Mental health professionals	Assessment schedule only
Sample G (3 papers)^a^	Infant behaviour program			BG		Clinical health workers	Standard pediatric primary care
Sample H (2 papers)^a^	Modified mother-infant transaction program			ER	X	Clinical health workers	Standard hospital protocol (e.g., infant screening, baby massage, discharge consultation) and follow-up care
Sample I (3 papers)^a^	Infant behavioral assessment and intervention program			ER	X	Clinical health workers	Received standard care and optional physical therapy
Sample J (5 papers)^a^	Mother-infant transaction program			ER	X	Clinical health workers	Routine nursery services
Sample K (2 papers)^a^	Toy demonstration program	X	X	ER		Mental health professionals	Toys delivered every week
Abikoff et al. (2015)	New forest parenting package			M		Mental health professionals	Received treatment of choice following completion of assessment schedule
Aboud and Akhter (2011)	Responsive feeding and stimulation	X	X	CR		Community volunteers	Received 12 informational sessions on health, nutrition and child development
Alvarenga et al. (2020)	Maternal sensitivity program			M		Other	Received magnets every month, showing the main behavioral acquisitions they should expect to observe in their babies
Bernard et al. (2017)	Attachment and biobehavioral catch-up			ER		Trained interventionists	Developmental education for families (DEF)
Boivin et al., (2013a)	Mediational intervention for sensitizing caregivers	X	X	CR		Other	Nutrition and hygiene curriculum
Boivin et al., (2013b)	Mediational intervention for sensitizing caregivers	X	X	CR		Trained interventionists	Nutrition and hygiene curriculum
Boivin et al. (2017)	Mediational intervention for sensitizing caregivers	X	X	CR		Other	Nutrition and hygiene curriculum
Cameron et al. (2021)	Growing: birth to three	X	X	ER	X	Educators	Regular follow-ups at the outpatient clinic and one home-visit one month after discharge from the hospital
Cassidy et al. (2017)	Circle of security parenting intervention			ER		Clinical health workers	Offered to attend the circle of security-P program following assessment schedule
Cicchetti et al. (2000)	Toddler-parent psychotherapy	X	X	ER		Mental health professionals	Assessment schedule only
Colditz et al. (2019)	Baby triple P for preterm infants			ER		Clinical health workers	Routine care for preterm infants
Cooper et al. (2015)	Index Intervention			ER		Clinical health workers	Routine primary care
Dubois-Comtois et al. (2017)	Attachment video-feedback intervention			ER		Clinical health workers	Received standard agency services, (i.e., monthly visit by a child welfare caseworker)
Elizur et al. (2017)	Hitkashrut			M	X	Mental health professionals	Minimal Intervention. Two consultation sessions using Hitkashrut's key components and handouts
Feeley et al. (2012)	Cues intervention			ER		Research staff	Attention control. Discussed topics related to newborn care and received information booklets
Feil et al. (2020)	Play and learning strategies (internet adaptation)			M		Trained interventionists	Attention control program
Fewell and Wheeden (1998)	Play and learn strategies	X		M		Research staff	Continued participation in public school program which included childcare curriculum
Flierman et al. (2016)	Transmural developmental support for preterm children and their parents (ToP) AND additive responsive parenting program (ToP +)			M		Mental health professionals	Usual care (which included participation in the ToP program during the first year)
Fong et al. (2019)	Phadthana khong dek			M		Clinical health workers	Assessment schedule only
Francis and Baker-Henningham (2021)	The irie homes toolbox			BG		Research staff	Waitlist control. Preschools were offered training in the Irie homes toolbox
Guttentag et al. (2014)	My baby & me	X	X	M		Trained interventionists	Low-intensity condition. Monthly phone calls from a coach, printed informational materials, and community resource referrals
Hutchings et al. (2017)	Incredible years toddler parenting programme			BG		Clinical health workers	Wait-list. Offered the intervention after the 6-month follow-up assessment
Jensen et al. (2021)	Sugira muryango			ER	X	Trained interventionists	Offered the social protection public works programme and other services as usual
Jin et al. (2007)	The mother's card			M		Mental health professionals	Assessment schedule only
Johnson et al. (2009)	Parent baby interaction programme			ER		Clinical health workers	Standard care in neonatal unit
Jones (2003)	Early childhood family check-up			BG		Mental health professionals	Wait-list control
Kersten-Alvarez et al. (2010)	Mother-baby intervention			ER		Trained interventionists	Three 15-min telephone calls providing information about child-rearing skills
Klein and Alony (1993)	Maternal mediation intervention	X		CR		Trained interventionists	Information on milestones in early development
Kyno et al. (2012)	Mother-infant transaction program			ER	X	Clinical health workers	Assessment schedule only
Landry et al. (2006)	Playing and learning strategies			M		Trained interventionists	Developmental feedback
Landry et al. (2017)	Play and learning strategies (in addition to head start)	X		M		Trained interventionists	Head start only
Landry et al. (2021)	Online play and learning strategies, ePALS (and head start)	X		M	X	Other	Head start only
Letourneau et al. (2011)	Peer support and maternal-infant interaction intervention			ER		Community volunteers	Two weeks of peer support after a 12-week waiting period
Madden et al. (1984)^b^	Mother–child home program	X	X	CR		Trained interventionists	Provision of toys and books
Madden et al. (1984)^c^	Mother–child home program	X	X	CR		Trained interventionists	Toys and books but no home sessions were provided
Madden et al. (1984)^d^	Mother–child home program	X	X	CR		Trained interventionists	Toys and books, but no home sessions were provided
Madden et al. (1984)^e^	Mother–child home program	X	X	CR		Trained interventionists	Toys and books, but no home sessions were provided
Magwaza and Edwards (1991)	An integrated parent-effectiveness and children's enrichment programme			CR		Research staff	Weekly two-hour home visits to discuss issues related to general community affairs
McManus et al. (2020)	Newborn behavioral observations (NBO) system			ER		Trained interventionists	Usual care. EI home-based service delivery, including therapeutic and developmental activities
Metzl et al. (1980)	Infant language program			M	X	Research staff	Assessment schedule only
Milgrom et al. (2013)	PremieStart-enhanced mother-infant transaction program			ER		Mental health professionals	Standard best-practice procedures for the care of preterm infants
Murray et al. (2016)	Home visiting intervention	X	X	ER		Community volunteers	Fortnightly visits by a community health worker who monitored maternal and infant health
Newnham et al. (2009)	Modified mother-infant transaction program			ER		Research staff	Standard hospital care
O'Bleness et al. (2015)	Child's game			M		Research staff	Play-as-usual. Eight play sessions once per week for 20 min each time
Pontoppidan (2020)	Incredible years parents and babies program			M	X	Trained interventionists	Usual care. Home visits, open consultation hours at a local clinic, voluntary participation in a social group, and extra support if needed
Roggman et al. (2009)	Bear river early head start program	X	X	ER		Educators	Assessment schedule only
Scarr and McCartney (1988)	Mother–child home program	X	X	CR		Trained interventionists	Assessment procedures only
Semenov et al. (2021)	R4R intervention and PEER(E)			M		Trained interventionists	Wait-list. Business as usual head start and early head start
Strayhorn and Weidman (1989)	Preventive mental health intervention			BG		Research staff	Minimal intervention. Shown the first two videotapes of the experimental group and received a copy of the parent handout
Tachibana et al. (2012)	Mother–child play activity program	X		CR		Other	Received play activity program after completion of assessment schedule
Walker et al. (2004)	Psychosocial intervention based on the programme for the enrichment of interactions between mothers and children	X	X	M		Clinical health workers	Infants were visited weekly to obtain information on infant feeding and morbidity

*ER* emotional responsiveness, *BG* behavioural guidance, *CR* cognitive responsiveness, *M* mixed; combination of 2 + targets

^a^Sample A (Clarke et al., 2012; Sheridan et al., 2011); Sample B (Jeong et al., 2019; Obradovic et al., 2016; Yousafzai et al., 2014; Yousafzai et al., 2016); Sample D (Brennan et al., 2013; Chang et al., 2015; Chang et al., 2017; Connell et al., 2019; Lunkenheimer et al., 2008); Sample E (Barrera et al., 1986; Barrera et al., 1991); Sample F (Green et al., 2015; Green et al., 2017); Sample G (Bagner et al., 2016; Garcia et al., 2019; Heymann et al., 2020); Sample H (Kaaresen et al., 2008; Nordhov et al., 2010); Sample I (Koldewijn et al., 2010; van Hus et al., 2013; Verkerk et al., 2012); Sample J (Achenbach et al., 1990, Achenbach et al., 1993; Nurcombe et al., 1984; Rauh et al., 1988; Rauh et al., 1990); Sample K (Slaughter et al., 1979;Slaughter et al., 1983)

^b^1973 Cohort

^c^1974 Cohort

^d^1975 Cohort

^e^1976 Cohort

#### Methodological Characteristics

Methodological characteristics are presented in Table 3. Studies spanned from 1979 to 2021. Including studies from overlapping samples, records were reported in journal articles (*k* = 74, 91.4%)**,** book chapters (*k* = 2, 2.5%), dissertations (*k* = 4, 4.9%), and a single working paper (1.2%). Sample sizes ranged from 29 to 1261 participants (median = 97, IQR = 57.50–184.00). Participants were recruited from 21 countries, most frequently the United States (*k* = 26, 42.6%). Interventions were delivered in the home (*k* = 26, 42.6%), home and one other setting (e.g., hospital or community; *k* = 14, 23%), hospital (*k* = 9, 14.8%), community (*k* = 4, 6.6%), research setting (*k* = 3, 4.9%), school (*k* = 3, 4.9%), community (*k* = 4, 6.6%), and via technology (*k* = 1, 1.6%).

**Table 3:** Methodological characteristics

Study	Record	Sample size	Setting	Country
Sample A (2 papers)^a^	1 Working paper 1 Journal article	217	Home	United States
Sample B (4 papers)^a^	4 Journal articles	1261	Home + other setting	Pakistan
Sample D (5 papers)^a^	5 Journal articles	645	Home	United States
Sample E (2 papers)^a^	2 Journal articles	45	Home	Canada
Sample F (2 papers)^a^	2 Journal articles	50	Home	UK
Sample G (3 papers)^a^	3 Journal articles	60	Home	United States
Sample H (2 papers)^a^	2 Journal articles	131	Hospital	Norway
Sample I (3 papers)^a^	2 Book chapters 1 Journal article	151	Hospital	Netherlands
Sample J (5 papers)^a^	5 Journal articles	74	Hospital	United States
Sample K (2 papers)^a^	2 Journal articles	40	Home + other setting	United States
Abikoff et al. (2015)	Journal article	101	Home	United States
Aboud and Akhter (2011)	Journal article	186	Community	Bangladesh
Alvarenga et al. (2020)	Journal article	44	Home	Brazil
Bernard et al. (2017)	Journal article	52	Home	United States
Boivin et al., (2013a)	Journal article	114	Research setting	Uganda
Boivin et al., (2013b)	Journal article	100	Home + other setting	Uganda
Boivin et al. (2017)	Journal article	221	Home + other setting	Uganda
Cameron et al. (2021)	Journal article	36	Home	Norway
Cassidy et al. (2017)	Journal article	141	School	United States
Cicchetti et al. (2000)	Journal article	97	Home + other setting	United States
Colditz et al. (2019)	Journal article	304	Hospital	Australia
Cooper et al. (2015)	Journal article	148	Home	UK
Dubois-Comtois et al. (2017)	Journal article	41	Home	Canada
Elizur et al. (2017)	Journal article	182	–^b^	Israel
Feeley et al. (2012)	Journal article	96	Hospital	Canada
Feil et al. (2020)	Journal article	150	Technological	United States
Fewell and Wheeden (1998)	Journal article	62	School	United States
Flierman et al. (2016)	Journal article	60	Home	Netherlands
Fong et al. (2019)	Dissertation	121	Home	Laos
Francis and Baker-Henningham (2021)	Journal article	212	–	Jamaica
Guttentag et al. (2014)	Journal article	225	Home + other setting	United States
Hutchings et al. (2017)	Journal article	89	Community	UK
Jensen et al. (2021)	Journal article	1084	Home	Rwanda
Jin et al. (2007)	Journal article	87	Home	China
Johnson et al. (2009)	Journal article	194	Hospital	UK
Jones (2003)	Dissertation	59	Research setting	United States
Kersten-Alvarez et al. (2010)	Journal article	58	Home	Netherlands
Klein and Alony (1993)	Journal article	59	Home	Israel
Kyno et al. (2012)	Journal article	57	Hospital	Norway
Landry et al. (2006)	Journal article	264	Home	United States
Landry et al. (2017)	Journal article	220	Home	United States
Landry et al. (2021)	Journal article	293	Home + other setting	United States
Letourneau et al. (2011)	Journal article	48	Home + other setting	Canada
Madden et al. (1984)^c^	Journal article	34	Home + other setting	United States
Madden et al. (1984)^d^	Journal article	48	Home + other setting	United States
Madden et al. (1984)^e^	Journal article	29	Home + other setting	United States
Madden et al. (1984)^f^	Journal article	55	Home + other setting	United States
Magwaza and Edwards (1991)	Journal article	60	Home	South Africa
McManus et al. (2020)	Journal article	38	–	United States
Metzl et al. (1980)	Journal article	40	Home	United States
Milgrom et al. (2013)	Journal article	91	Hospital	Australia
Murray et al. (2016)	Journal article	263	Home	South Africa
Newnham et al. (2009)	Journal article	63	Hospital	Australia
O'Bleness et al. (2015)	Dissertation	155	Research setting	United States
Pontoppidan et al. (2020)	Journal article	81	Home + other setting	Denmark
Roggman et al. (2009)	Journal article	161	Home	United States
Scarr and McCartney (1988)	Journal article	117	Home	Bermuda
Semenov et al. (2021)	Dissertation	128	–	United States
Strayhorn and Weidman (1989)	Journal article	95	–	United States
Tachibana et al. (2012)	Journal article	219	Home + other setting	Japan
Walker et al. (2004)	Journal article	131	Home	Jamaica

^a^Sample A (Clarke et al., 2012; Sheridan et al., 2011); Sample B (Jeong et al., 2019; Obradovic et al., 2016; Yousafzai et al., 2014; Yousafzai et al., 2016); Sample D (Brennan et al., 2013; Chang et al., 2015; Chang et al., 2017; Connell et al., 2019; Lunkenheimer et al., 2008); Sample E (Barrera et al., 1986; Barrera et al., 1991); Sample F (Green et al., 2015; Green et al., 2017; Sample G: Bagner et al., 2016; Garcia et al., 2019; Heymann et al., 2020); Sample H (Kaaresen et al., 2008; Nordhov et al., 2010); Sample I (Koldewijn et al., 2010; van Hus et al., 2013; Verkerk et al., 2012); Sample J (Achenbach et al., 1990, Achenbach et al., 1993; Nurcombe et al., 1984; Rauh et al., 1988; Rauh et al., 1990); Sample K (Slaughter et al., 1979;Slaughter et al., 1983)

^b^Missing data

^c^1973 Cohort

^d^1974 Cohort

^e^1975 Cohort

^f^1976 Cohort

#### Risk of Bias

**Fig. 2:**
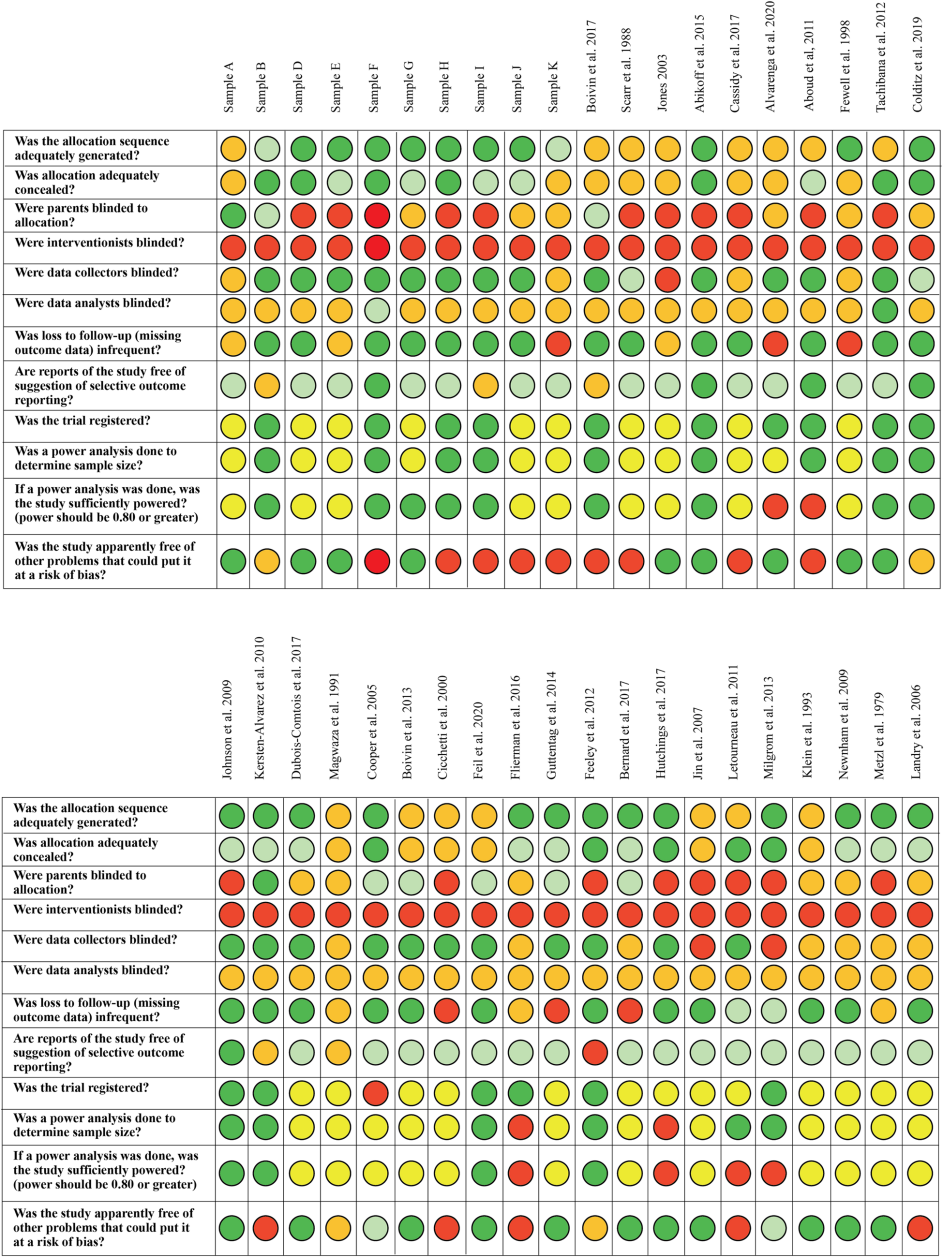

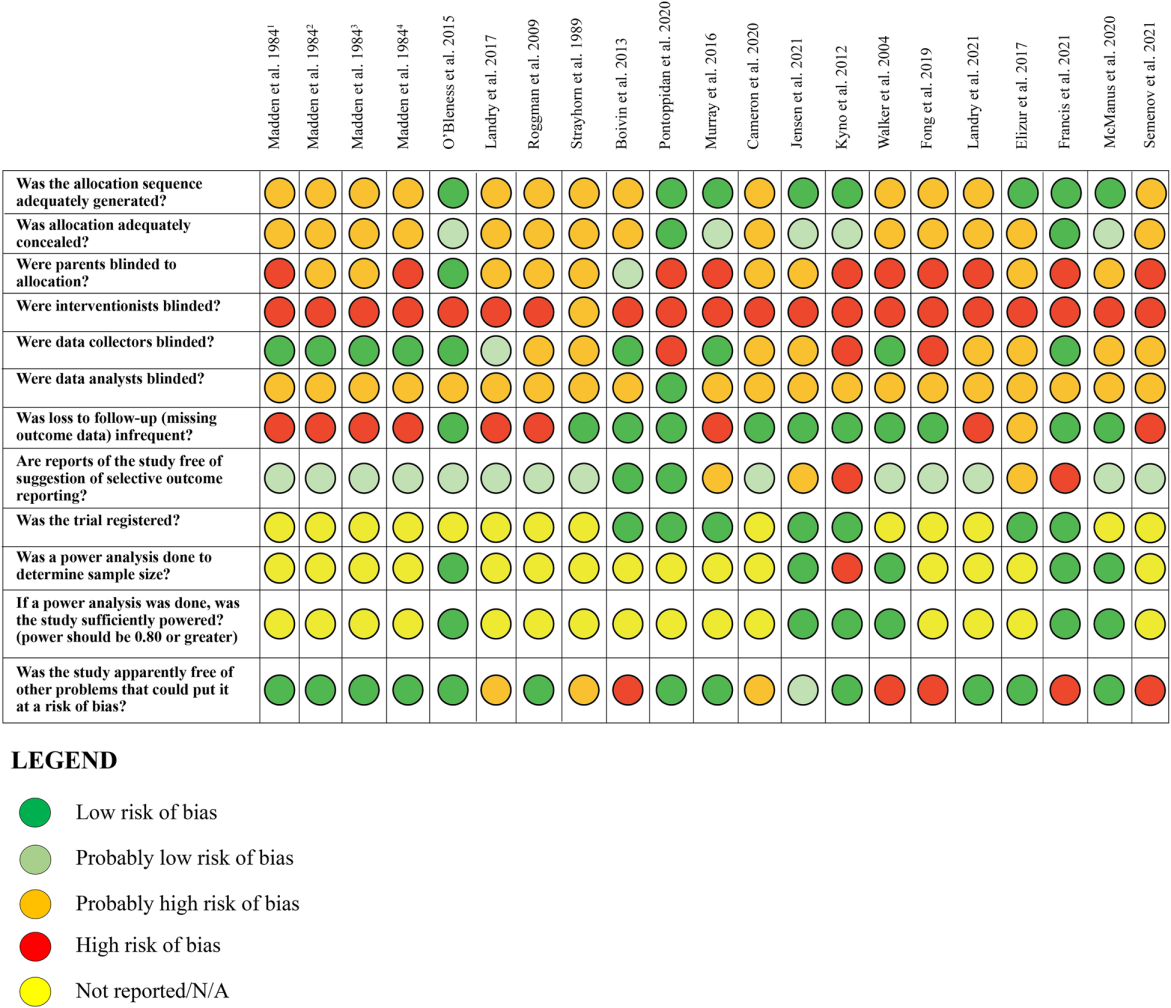
Clarity tool: risk of bias assessment. One optional question is not included due to a large amount of missing data: ‘Masking Questions E: Other groups masked to group allocation’. ^1^1973 cohort, ^2^1974 cohort, ^3^1975 cohort, ^4^1976 cohort

The risk of bias assessment for individual studies is presented in Fig. 2. Summary descriptive information for each criterion is provided in Table 4. Based on ratings of *probably yes* or *definitely yes*, many studies reported adequate allocation sequence generation (*k* = 33; 54.1), allocation concealment (*k* = 33; 54.1%), and use of masked data collectors (*k* = 36; 59%), thus mitigating potential selection and detection biases. Most studies reported low attrition rates (*k* = 39; 64%) and did not report other biases such as significant baseline differences (*k* = 35; 57.4%). Regarding selective outcome reporting, most studies (*k* = 50; 82%) showed consistency in the outcomes reported in their methods and results sections. However, given that a minority of studies reported trial registration information (*k* = 23, 37.7%), selective outcome reporting was mostly rated as *probably* low risk, as we could not confirm planned analyses. Twenty studies (32.8%) reported a priori power analyses to determine required sample sizes. Further, due to the nature of intervention-based studies, those delivering the parenting intervention could not be masked to participant group allocation (no studies with masked interventionists), and participants were not commonly masked (typically when two active treatments were compared to a control group*; k* = 11; 18.0%). Finally, three studies (4.9%) were rated as having masked data analysts, due to infrequent reporting on this item. Total risk of bias scores ranged from 0 to 9 (mean score = 4.95).

**Table 4:** Risk of bias summary

	Definitely yes *f*(%)	Probably yes *f*(%)	Probably no *f*(%)	Definitely no *f* (%)
1. Allocation sequence adequately generated?	31 (50.8)	2 (3.3)	28 (45.9)	–
2. Allocation adequately concealed?	14 (23)	19 (31.1)	28 (45.9)	–
3. Parents masked to allocation?	3 (4.9)	8 (13.1)	21 (34.4)	29 (47.5)
4. Interventionists masked?	–	–	1 (1.6)	60 (98.4)
5. Data collectors masked?	33 (54.1)	3 (4.9)	19 (31.1)	6 (9.8)
6. Data analysts masked?	2 (3.3)	1 (1.6)	58 (95.1)	–
7. Loss to follow-up infrequent?	37 (60.7)	2 (3.3)	7 (11.5)	15 (24.6)
8. Free of selective outcome reporting?	7 (11.5)	43 (70.5)	8 (13.1)	3 (4.9)
9. Trial registered?	23 (37.7)	–	37 (60.7)	1 (1.6)
10. Power analysis completed?	20 (32.8)	–	38 (62.3)	3 (4.9)
11. Study sufficiently powered?	19 (31.1)	–	36 (59.0)	6 (9.8)
12. Free of other problems?	32 (52.5)	3 (4.9)	7 (11.5)	19 (31.1)

### Meta-Analyses

#### Mental Abilities

**Fig. 3:**
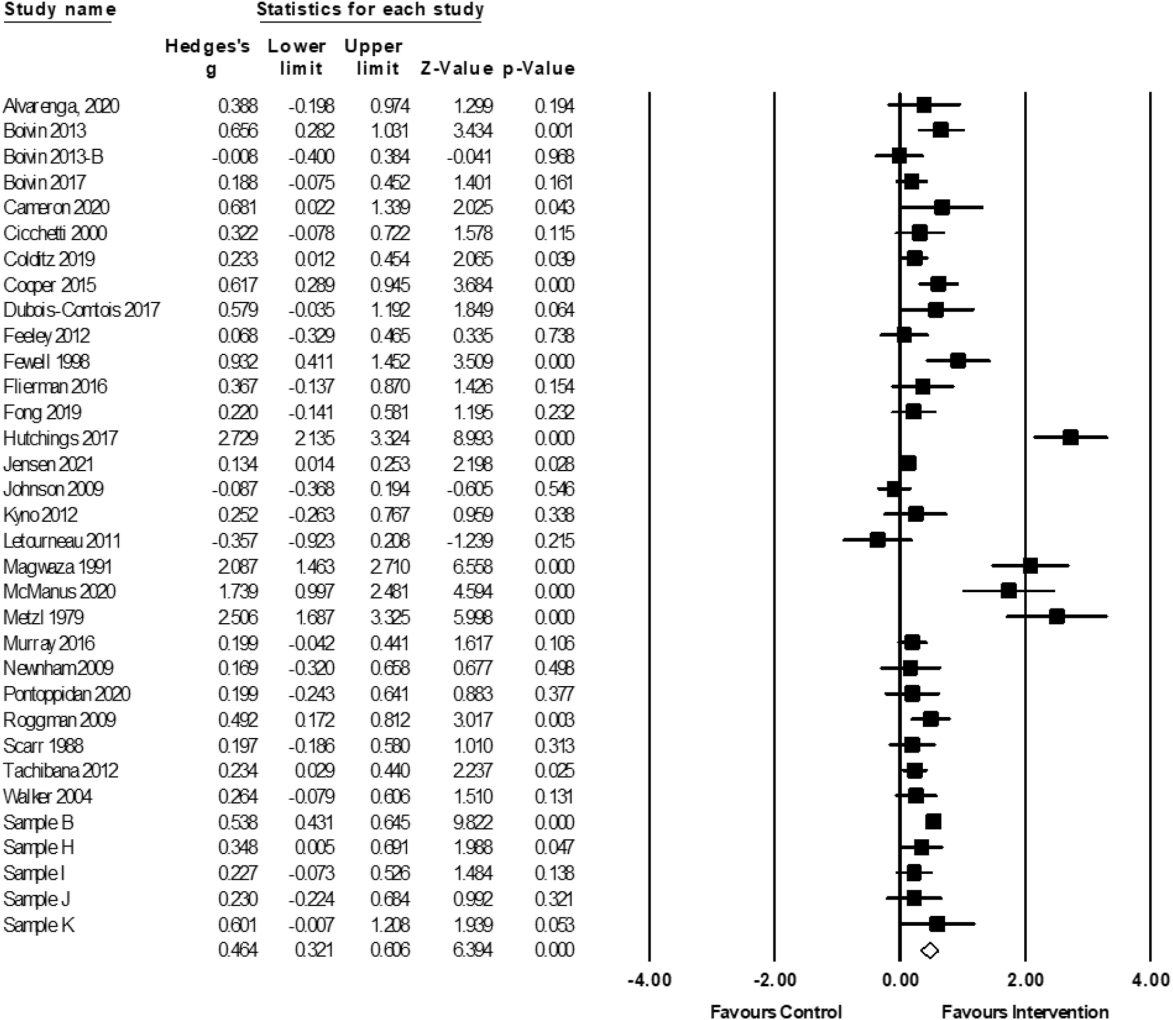
Forest plot for meta-analysis of mental abilities outcomes (*k* = 33). Sample B (Jeong et al., 2019; Obradovic et al., 2016; Yousafzai et al., 2014; Yousafzai et al., 2016); Sample H (Kaaresen et al., 2008; Nordhov et al., 2010); Sample I (Koldewijn et al., 2010; van Hus et al., 2013; Verkerk et al., 2012); Sample J (Achenbach et al., 1990, Achenbach et al., 1993; Nurcombe et al., 1984; Rauh et al., 1988; Rauh et al., 1990); Sample K (Slaughter et al., 1979; Slaughter et al., 1983)

Meta-analytic findings for all four meta-analyses, and sensitivity analyses, can be found in Table 5. Positive parenting interventions led to significant improvements in children’s mental abilities, based on 5746 participants (*g* = 0.46; 95% CI 0.32, 0.61, *p* < 0.0001, *k* = 33; Fig. 3). Five samples with missing data were excluded from analyses using listwise deletion (sensitivity analyses below).^4^ The Duval and Tweedie’s trim-and-fill procedure did not provide evidence of publication bias (i.e., no additional effect sizes were imputed to balance reported positive intervention effects).

**Table 5:** Meta-analytic results and sensitivity analyses for all outcomes

	Main analysis				Sensitivity analyses								
					Pre-post correlation		Combining outcomes		Outcome assessment		Missing data		
	*k*	*n*	*g*	*Q*	*g*	*Q*	*g*	*Q*	*g*	*Q*	*k*	*g*	*Q*
Mental Abilities	33	5746	0.46**	197.62**	0.43**	171.83**	0.47**	197.24**	0.43**	182.51**	38	0.41**	210.02**
Language	30	6248	0.25**	117.63**	0.24**	98.42**	0.25**	110.88**	0.25**	75.40**	34	0.23**	119.45**
Exec. Functions	14	3628	0.07	74.88**	0.07	57.51**	0.07	53.18**	0.06	70.24**	14	0.07	74.88**
Pre-Academics	7	2365	0.16^†^	29.89**	0.14^†^	21.04^*^	0.16^†^	21.2^*^	0.16^†^	29.89**	8	0.14^†^	33.55**

**p < 0.01; *p < 0.05; ^†^*p* < 0.1

**Fig. 4:**
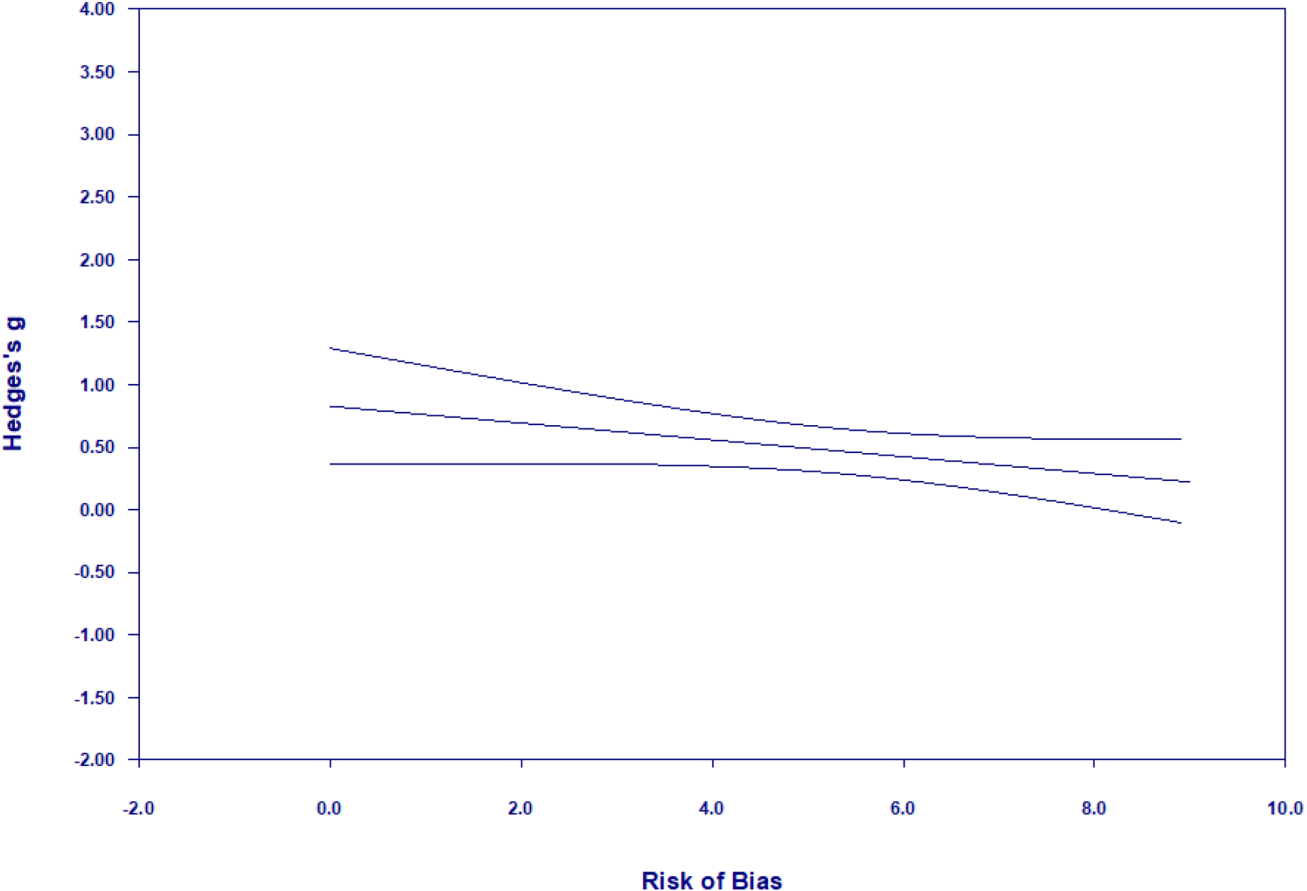
Meta-regression of Hedge’s g on risk of bias for mental abilities meta-analysis. Higher scores on risk of bias represents lower risk of bias

Significant heterogeneity (*Q*(32) = 197.62, *p* < 0.0001) and true dispersion (*I*^2^ = 83.81) were evident. Moderation results are presented in Table 6. Only significant findings are reported in-text, but several near-significant moderators emerged and can be seen in Tables 6, 7, 8. Studies that were scored as having a higher risk of bias yielded larger effect sizes than those that were rated as having a lower risk of bias (*β* =  − 0.07, CI − 0.13, − 0.003, *p* < 0.05, *z* = − 2.07; Fig. 4). Furthermore, larger improvements in mental abilities were detected amongst studies that used standardized direct assessments of child mental abilities (*g* = 0.53; 95% CI 0.36, 0.70, *k* = 27), as compared to those that used parent-reported outcome measures (*g* = 0.16; 95% CI 0.06, 0.26, *k* = 6).

**Table 6:** Results of moderator analyses for meta-analysis of mental abilities (*k* = 33)

	Categorical moderators				
	*k*	*g*	95% CI lower	95% CI upper	Q
Child characteristics					
Child birth risk	–	–	–	–	3.18^†^
None reported	20	0.57	0.35	0.80	
Some reported	13	0.32	0.28	0.55	
Child emotional-behavioural risk	–	–	–	–	–
None reported	31	–	–	–	
Some reported	2	–	–	–	
Contextual characteristics					
Parental education	–	–	–	–	0.85
Mid/High	17	0.58	0.30	0.85	
Low	14	0.42	0.24	0.60	
Household income	–	–	–	–	0.09
Mid/High	16	0.52	0.27	0.76	
Low	16	0.47	0.28	0.65	
Parental mental health	–	–	–	–	3.69^†^
None reported	28	0.52	0.36	0.68	
Some reported	5	0.22	-0.05	0.48	
Intervention characteristics					
Intervention type	–	–	–	–	2.27
Behavioural guidance	1	–	–	–	
Cognitive responsiveness	6	0.49	0.11	0.87	
Emotional responsiveness	19	0.32	0.18	0.45	
Mixed (1 + of above)	7	0.61	0.20	1.02	
Intensity	–	–	–	–	1.85
< 16 sessions	20	0.56	0.32	0.80	
16 + sessions	13	0.37	0.24	0.50	
Duration	–	–	–	–	2.17
< 12 months	22	0.55	0.33	0.77	
12 + months	11	0.36	0.22	0.49	
Father involvement	–	–	–	–	0.05
None	25	0.48	0.31	0.65	
Some	8	0.44	0.15	0.73	
Methodological characteristics					
Publication status	–	–	–	–	–
Published	32	–	–	–	
Unpublished	2	–	–	–	
Instrument measure	–	–	–	–	13.25**
Observation	0	–	–	–	
Report	6	0.16	0.06	0.26	
Standardized/direct assessment	27	0.53	0.36	0.70	

	Continuous moderators				
	*k*	*β*	95% CI lower	95% CI upper	*z* score
Substantive moderators					
Child age, baseline	33	0.005	− 0.004	0.01	1.04
Child age, outcome assessment	33	− 0.001	− 0.01	0.01	− 0.19
Child sex, male	33	0.01	− 0.02	0.05	0.77
Parental age	29	− 0.01	− 0.05	0.02	− 0.65
Methodological moderators					
Year	33	− 0.013	− 0.03	0.0001	− 1.95^†^
Sample size	33	− 0.0003	− 0.001	0.0002	− 1.02
Risk of bias, total score	33	− 0.07	− 0.13	− 0.01	− 2.07^*^

**p < 0.01; *p < 0.05; ^†^*p* < 0.1

When statistically significant moderators (risk of bias scores and method of assessment) were simultaneously entered into a meta-regression, risk of bias was significant (*β* =  − 0.07, CI − 0.13, − 0.004, *p* < 0.05, *z* = − 2.09), and instrument measurement was reduced to *p* < 0.10 (*β* =  − 0.32, CI − 0.70, 0.06, *z* =  − 1.64).

#### Language

**Fig. 5:**
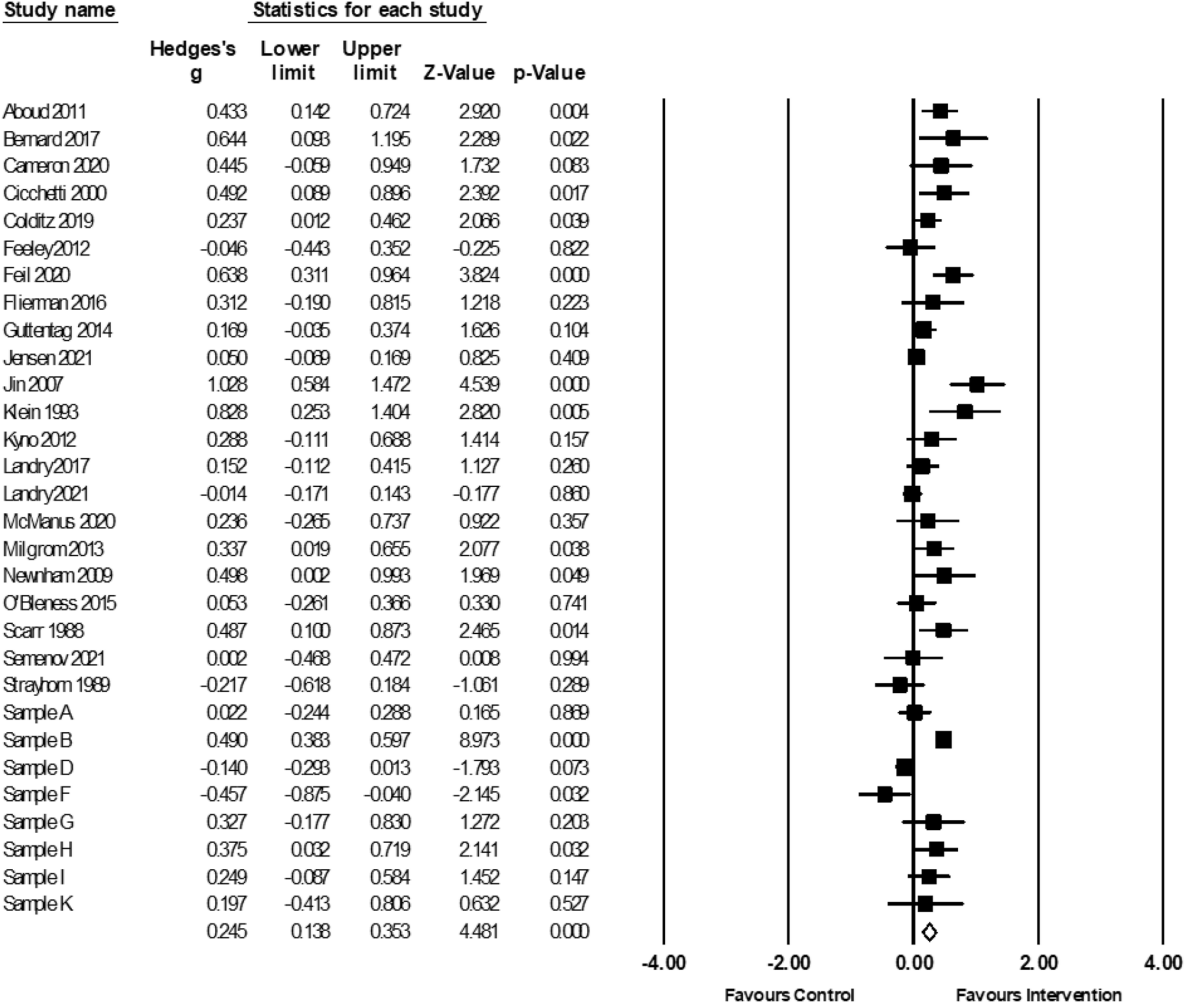
Forest plot for meta-analysis of language outcomes (*k* = 30). Sample A (Clarke et al., 2012; Sheridan et al., 2011); Sample B (Jeong et al., 2019; Obradovic et al., 2016; Yousafzai et al., 2014; Yousafzai et al., 2016); Sample D (Brennan et al., 2013; Chang et al., 2015; Chang et al., 2017; Connell et al., 2019; Lunkenheimer et al., 2008); Sample F (Green et al., 2015; Green et al., 2017); Sample G (Bagner et al., 2016; Garcia et al., 2019; Heymann et al., 2020); Sample H (Kaaresen et al., 2008; Nordhov et al., 2010); Sample I (Koldewijn et al., 2010; van Hus et al., 2013; Verkerk et al., 2012); Sample K (Slaughter et al., 1979; Slaughter et al., 1983)

**Fig. 6:**
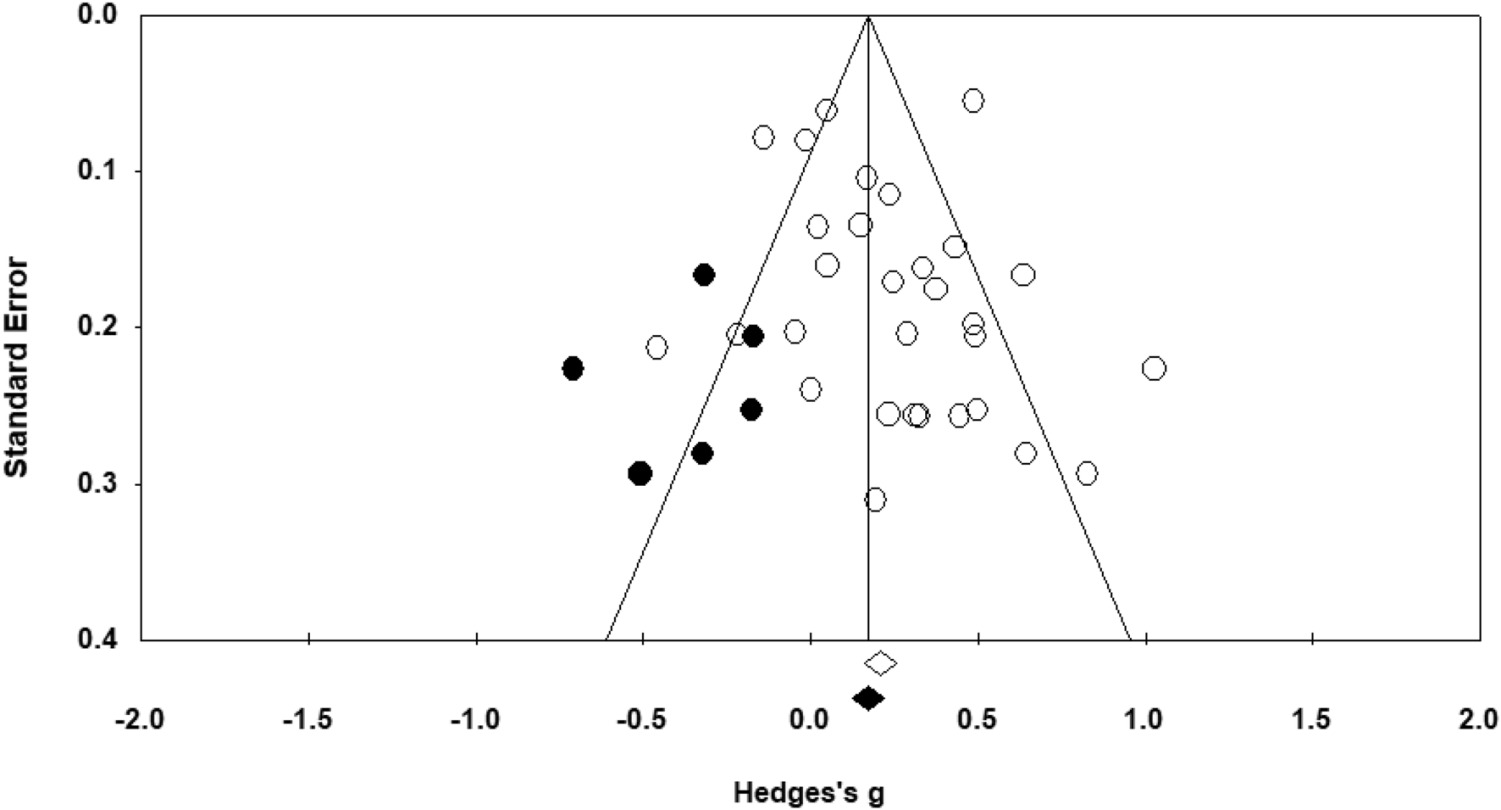
Funnel plot of observed and imputed studies for language meta-analysis

Based on a meta-analysis of 30 studies (*n* = 6248), positive parenting interventions led to significant gains in children’s language skills (*g* = 0.25, 95% CI 0.14, 0.35, *p* < 0.0001; Fig. 5). Four samples with missing data were excluded from analyses. Duval and Tweedie’s trim-and-fill procedure indicated six studies imputed to balance reported positive intervention effects, based on random effects models (adjusted point estimate = 0.16 (95% CI 0.05, 0.27; Fig. 6). Rosenthal's (1991) *Fail-safe N* was examined, as an additional indicator of potential publication bias, which indicated that 516 studies with null results would be required to reduce the *p*-value to below significance.

**Fig. 7:**
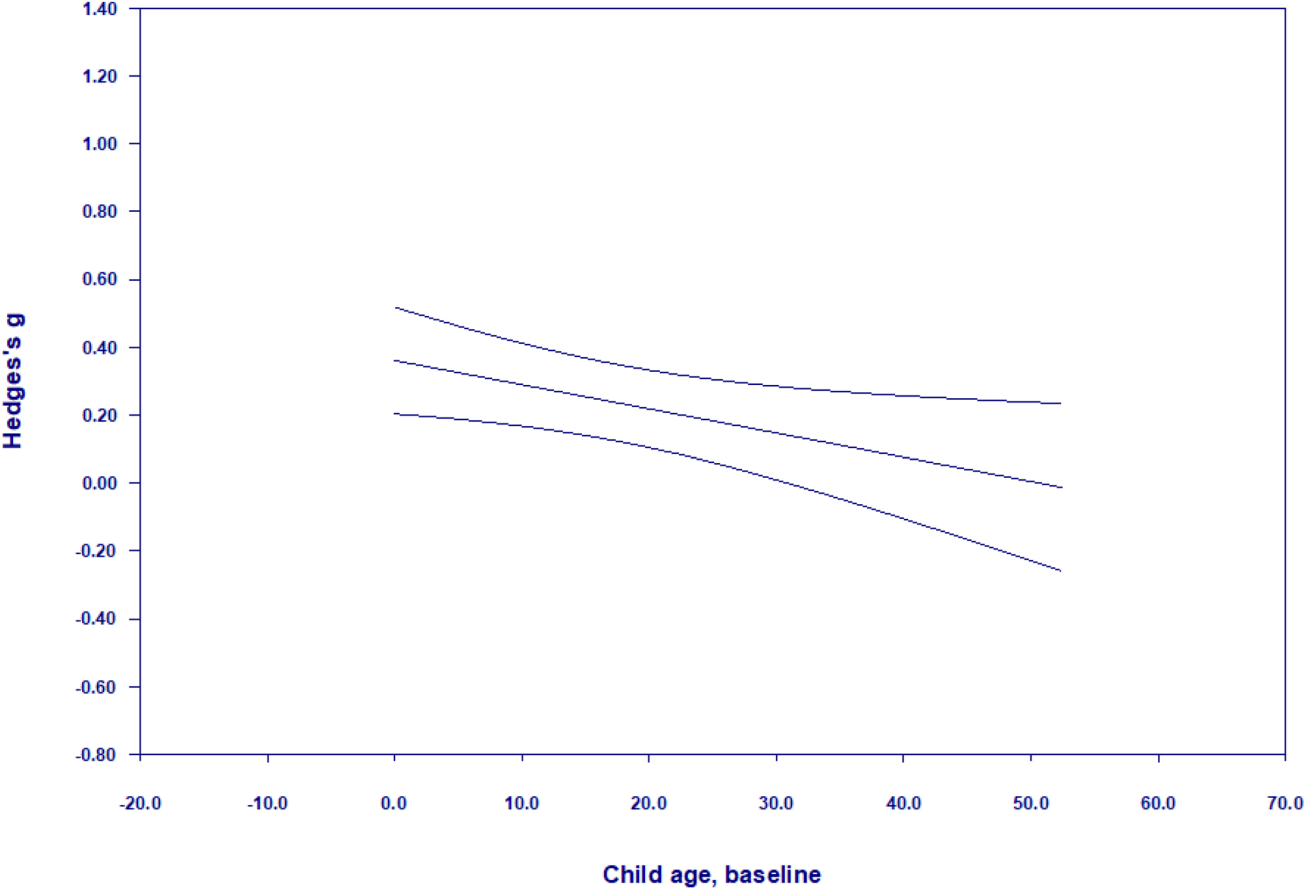
Meta-regression of Hedge’s g on child age (at baseline) for language meta-analysis

There was significant heterogeneity (*Q*(29) = 117.63, *p* < 0.0001), with evidence of true dispersion I^*2*^ = 75.35. Moderation analyses, presented in Table 7, showed that younger children made more gains than older children (β = − 0.01, CI − 0.01, − 0.002, *p* < 0.01, *z* = − 2.78; Fig. 7). Interventions were more effective with parents who had more than a high school education (*g* = 0.36; 95% CI 0.23, 0.49, *k* = 15) as compared to those with less than or equal to high school education (*g* = 0.14; 95% CI − 0.01, 0.30, *k* = 15). Furthermore, interventions that only included mothers (*g* = 0.29; 95% CI 0.16, 0.41, *k* = 23) were more effective than those that also included fathers (*g* = 0.09; 95% CI − 0.04, 0.23, *k* = 7).

**Table 7:** Results of moderator analyses for meta-analysis of language (k = 30)

	Categorical moderators				
	*k*	*g*	95% CI lower	95% CI upper	Q
Child characteristics					
Child birth risk	–	–	–	–	3.19^†^
None reported	19	0.21	0.07	0.34	
Some reported	11	0.36	0.26	0.45	
Child emotional-behavioural risk	–	–	–	–	–
None reported	26	–	–	–	
Some reported	4	–	–	–	
Contextual characteristics					
Parental education	–	–	–	–	4.32*
Mid/High	15	0.36	0.23	0.49	
Low	15	0.14	− 0.01	0.30	
Household income	–	–	–	–	2.02
Mid/High	12	0.32	0.22	0.43	
Low	18	0.19	0.04	0.34	
Parental mental health	–	–	–	–	0.64
None reported	25	0.27	0.16	0.38	
Some reported	5	0.09	− 0.33	0.51	
Intervention characteristics					
Intervention type	–	–	–	–	.07
Behavioural guidance	3	–	–	–	–
Cognitive responsiveness	3	–	–	–	–
Emotional responsiveness	15	0.26	0.12	0.41	
Mixed (1 + of above)	9	0.23	0.04	0.42	
Intensity	–	–	–	–	0.28
< 16 sessions	19	0.22	0.09	0.35	
16 + sessions	11	0.29	0.10	0.47	
Duration	–	–	–	–	0.11
< 12 months	21	0.23	0.11	0.35	
12 + months	9	0.27	0.06	0.49	
Father involvement	–	–	–	–	4.19*
None	23	0.29	0.16	0.41	
Some	7	0.09	− 0.04	0.23	
Methodological characteristics					
Publication status	–	–	–	–	–
Published	27	–	–	–	
Unpublished	3	–	–	–	
Instrument measure	–	–	–	–	0.21
Report/observation	9	0.28	0.12	0.44	
Standardized/direct assessment	21	0.23	0.09	0.37	

	Continuous moderators				
	*k*	*β*	95% CI lower	95% CI upper	*z* score
Substantive moderators					
Child age, baseline	30	− 0.01	− 0.01	0.00	− 2.78**
Child age, outcome assessment	30	0.00	− 0.01	0.00	− 1.03
Child sex, male	29	0.00	− 0.02	0.03	0.38
Parental age	26	0.00	− 0.03	0.02	− 0.10
Methodological moderators					
Year	30	− 0.01	− 0.02	0.006	− 0.95
Sample size	30	− 0.0001	− 0.0004	0.0003	− 0.51
Risk of bias, total score	30	− 0.01	− 0.05	0.04	− 0.34

**p < 0.01; *p < 0.05; ^†^*p* < 0.1

When child age (at baseline), parental education, and father involvement were entered into a meta-regression simultaneously, child age at baseline (*β* = − 0.01, CI − 0.01, − 0.0003, *z* = − 1.85) was reduced to near-significance (*p* = 0.06), whereas parental education and father involvement were no longer significant moderators.

#### Executive Functioning

**Fig. 8:**
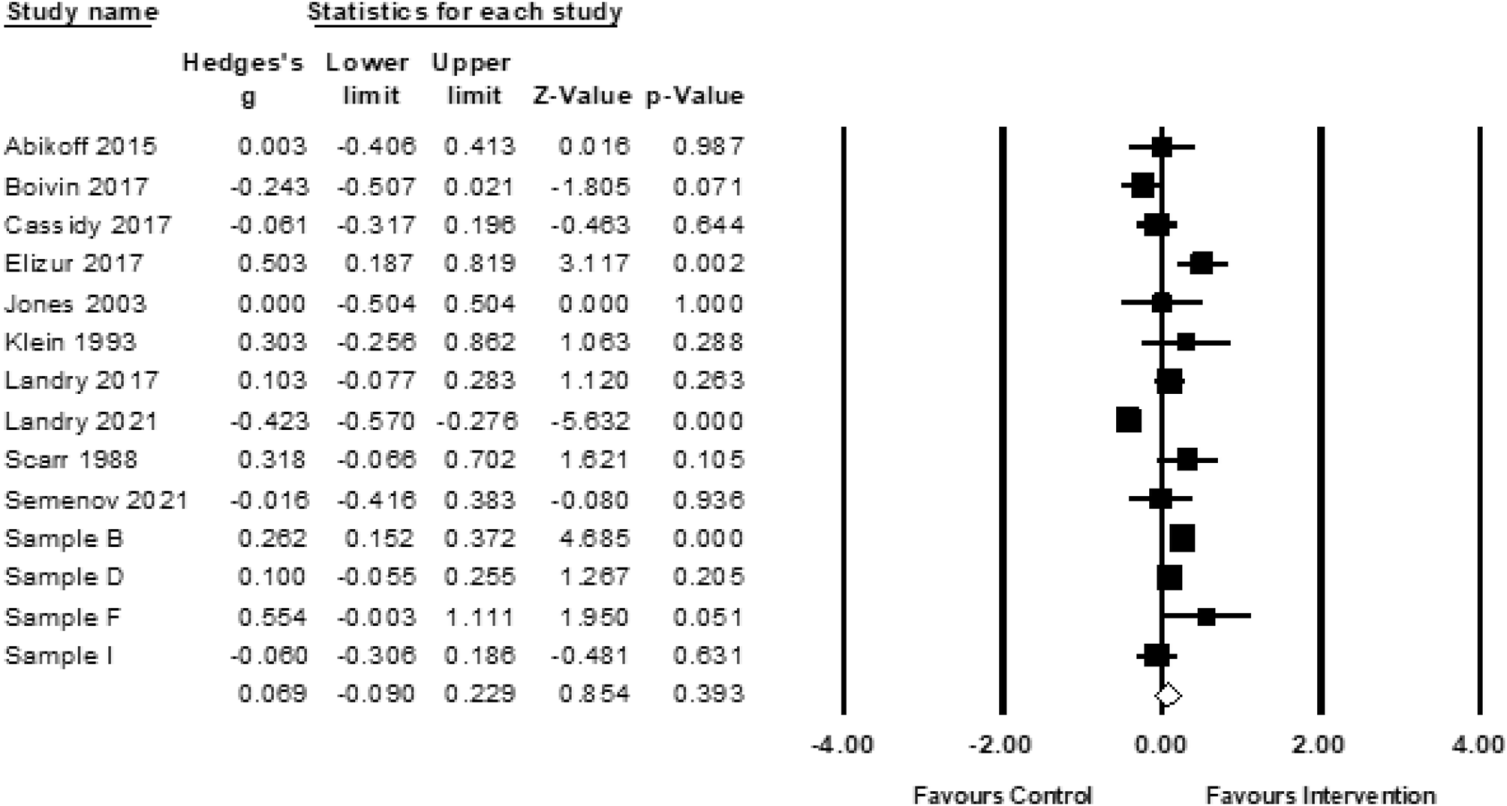
Forest plot for meta-analysis of executive functioning outcomes (*k* = 14). Sample B (Jeong et al., 2019; Obradovic et al., 2016; Yousafzai et al., 2014; Yousafzai et al., 2016); Sample D (Brennan et al., 2013; Chang et al., 2015; Chang et al., 2017; Connell et al., 2019; Lunkenheimer et al., 2008); Sample F (Green et al., 2015; Green et al., 2017); Sample G (Bagner et al., 2016; Garcia et al., 2019; Heymann et al., 2020); Sample I (Koldewijn et al., 2010; van Hus et al., 2013; Verkerk et al., 2012)

There were 14 studies (*n* = 3628) included in the meta-analysis of executive functioning outcomes, with results indicating a nonsignificant pooled effect size (*g* = 0.07; 95% CI − 0.09, 0.23, *ns;* Fig. 8). The Duval and Tweedie’s trim and fill procedure did not provide evidence of publication bias.

There was evidence of significant heterogeneity (*Q*(13) = 74.88, *p* < 0.0001), including true dispersion (*I*^2^ = 82.64). Categorical moderators were not examined due to small cell sizes (i.e., < 5 studies per cell), except for intervention intensity, with cell sizes allowing for moderation analysis. Intervention intensity did not emerge as a significant moderator. As shown in Table 8, only one continuous moderator emerged to explain between-study variability; parental age was negatively associated with effect size, wherein interventions that included samples of younger mothers had larger effect sizes than those with older mothers (β = − 0.04, CI − 0.08, − 0.003, *p* < 0.05, *z* = 2.11). To examine the potential confounding effect of child age, we included child and parental age in a meta-regression simultaneously. When controlling for baseline child age, parental age was reduced to *p* < 0.10.

**Table 8:** Results of moderator analyses for meta-analysis of executive functioning (*k* = 14)

Continuous moderators	k	β	95% CI Lower	95% CI Upper	z score
Substantive moderators					
Child age, baseline	14	− 0.005	− 0.01	0.003	− 1.18
Child age, outcome assessment	14	− 0.01	− 0.02	0.01	− 1.17
Child sex, male	14	0.01	− 0.01	0.03	1.1
Parental age	11	− 0.04	− 0.08	− 0.003	− 2.11*
Methodological moderators					
Year	14	− 0.01	− 0.03	0.01	− 1.27
Sample size	14	0.0001	− 0.0003	0.001	0.44
Risk of bias, total score	14	0.02	− 0.03	0.08	0.78

**p < 0.01; *p < 0.05; ^†^*p* < 0.1

#### Pre-Academics

**Fig. 9:**
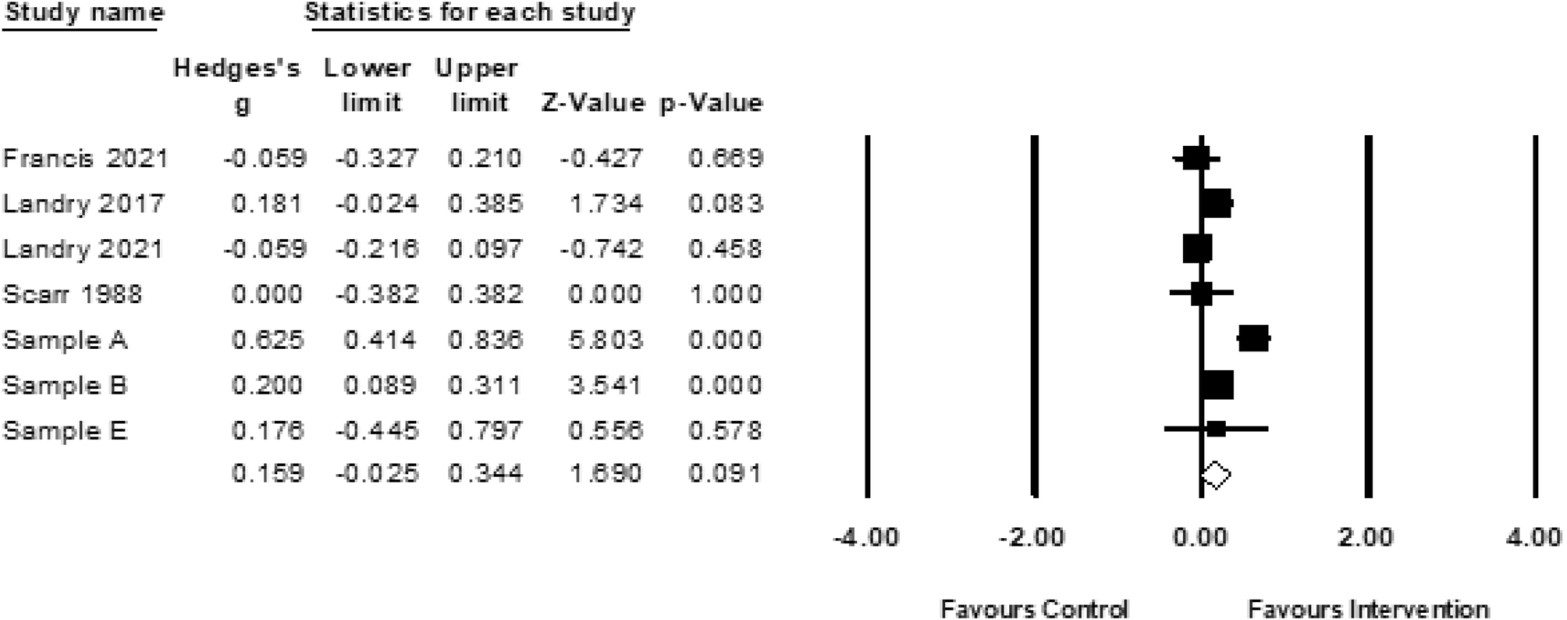
Forest plot for meta-analysis of pre-academic outcomes (*k* = 7). Sample A (Clarke et al., 2012; Sheridan et al., 2011); Sample B (Jeong et al., 2019; Obradovic et al., 2016; Yousafzai et al., 2014; Yousafzai et al., 2016); Sample E (Barrera et al., 1986; Barrera et al., 1991)

A meta-analysis of studies that included an outcome assessment of pre-academics (*k* = 7; *n* = 2365) yielded a positive but nonsignificant pooled effect (*g* = 0.16; 95% CI − 0.03 0.34, *p* < 0.10; Fig. 9). One sample with missing data was excluded from analyses. The Duval and Tweedie’s trim-and-fill procedure did not provide evidence of publication bias (i.e., no additional effect sizes were imputed). There was significant heterogeneity (*Q*(5) = 29.89, *p* < 0.01) and dispersion (*I*^2^ = 79.93). However, limited studies precluded moderation analyses.

### Sensitivity Analyses

Sensitivity analyses were conducted independently for each of the four meta-analyses. First, a sensitivity analysis for the imputation of pre-post score correlations was conducted, when not available (default = 0.7; sensitivity analysis = 0.5). Second, we examined the sensitivity of findings to our method of adjusting for the imputed correlations amongst outcome variables when combining effect sizes (default = 0.2; sensitivity analysis = 0.7). Third, analyses were conducted wherein the latest available outcome assessment was included (rather than earliest). Finally, analyses were conducted wherein 0.0 was substituted for missing values where data were not available for the calculation of standardized difference scores. Across sensitivity analyses, results of the pooled point estimates did not substantively change (Table 5). Thus, meta-analytic results appear robust.

## Discussion

The aim of the current systematic review and meta-analysis was to synthesize the extant literature on RCTs of positive parenting interventions that include an outcome assessment of child cognition in children under age six. Sixty-one studies from independent samples, spanning 1979 to 2021, were included in the review, yielding diverse methodologies related to sample, intervention, and publication characteristics. Findings highlight variability in effect sizes as a function of outcome domain, with mental abilities and language analyses yielding positive and significant pooled effect sizes, and executive functioning and pre-academics yielding smaller and nonsignificant pooled effect sizes. Outcome-specific moderation analyses in the mental abilities and language meta-analyses illustrate important conditions under which positive parenting interventions yield the strongest effects. Findings are considered robust based on a series of four sets of sensitivity analyses, which derived similar results in every case.

### Outcome Domains

Positive parenting interventions were effective in enhancing mental abilities and language. Children whose parents were assigned to a positive parenting intervention made mental gains that were close to half (*g* = 0.46) of a standard deviation higher, and language gains that were a quarter (*g* = 0.25) of a standard deviation higher, than those whose parents were assigned to a control group. This contrasts with executive functioning and pre-academic outcomes, where the pooled effect sizes were smaller and nonsignificant. This pattern of findings is consistent with the recent review by Jeong et al. (2021), wherein moderate effect sizes were found for mental abilities and language, with smaller effects for behaviour problems, which show significant overlap with executive functioning (Schoemaker et al., 2013). The differences in effect sizes amongst outcomes are substantial and likely meaningful, further elaborated below.

The effect of positive parenting interventions on pre-academic skills was positive but nonsignificant. Positive parenting programs may not directly influence children’s pre-academic skills. Parenting interventions that focus on home-literacy activities and skill development may confer greater benefits to children’s developing literacy and numeracy skills, though this remains speculative. As a possible alternative, the fewer studies in the pre-academic analyses (*k* = 7) may have affected power and precision of effect estimates. As the number of primary studies grows, future meta-analyses will be informative, as will an examination of potential moderators of intervention effectiveness.

There was no evidence of positive parenting programs enhancing executive functioning skills. Positive parenting programs may not directly influence children’s developing executive processes. Given that executive functioning is highly heritable (Miyake & Friedman, 2012), it is important to consider whether it is influenced by normative variations in positive parenting. Rather, it may be that early executive functioning is more dependent on experiences of severe environmental adversity, such as threat (e.g., abuse, exposure to IPV) and/or deprivation (i.e., neglect, institutional rearing, and food insecurity Johnson et al., 2021; Zelazo, 2020). Alternatively, there may be undetected timing effects operating. For instance, only four of the 14 studies examining executive functioning as an outcome included interventions prior to child age 24 months. However, of the three studies that showed positive and significant effects, two were initiated prior to child age 24 months (Green et al., 2015; Obradović et al., 2016), and one was initiated at 48 months (Elizur et al., 2017). This provides some preliminary evidence that interventions initiated earlier may be more effective, which is in line with findings from the language meta-analysis (elaborated below) and other psychosocial interventions assessing cognitive recovery (Baudry et al., 2017; Castle et al., 1999; Nelson et al., 2007). In the future, with a larger number of primary studies, timing effects and social disadvantage, as well as interactions between these variables, should be examined as moderators of intervention effectiveness.

There are two additional considerations regarding differential patterns of effectiveness. First, language and mental abilities are easily (and typically) assessed by well-validated, extensively used standardized direct assessments. In contrast, definitions, operationalization, and measurement vary widely across methods of assessment of executive functioning and pre-academics. Furthermore, whereas mental abilities and language emerge in the first two years of life, more complex executive functions and pre-academic skills emerge in toddlerhood and beyond. Thus, the latter constructs are particularly difficult to assess in the earlier years, as nascent skills are only beginning to emerge. As a result, observed differences in effect sizes may, in part, be due to differences in measurement, particularly early in development. Finally, it is helpful to consider the nature of content in positive parenting interventions. Specifically, most studies targeted emotional responsiveness (*k* = 25), with only six studies targeting behavioural guidance and an additional 12 targeting cognitive responsiveness. It may be that executive functioning and pre-academics are better targeted by programs that encourage behavioural guidance and/or cognitive responsiveness. Indeed, as mentioned, positive parenting is a non-specific term that includes varied, though overlapping, behaviours. Given this, future research needs to identify those aspects of positive parenting that are most powerfully linked to different cognitive outcomes.

Importantly, gains made in mental abilities and language following positive parenting interventions may have positive cascading effects on executive functioning and/or pre-academic skills. For instance, language abilities and executive functioning are reciprocally related in early childhood (Romeo et al., 2022; Xing et al., 2021), and children’s language and nonverbal problem-solving skills mediate children’s later executive processes (Landry et al., 2002). Indeed, in a study included in the current review, though no direct effects were observed for executive functioning following a positive parenting intervention, positive changes to language functioning led to subsequent gains in executive functioning (Chang et al., 2015). Relatedly, children's aptitudes for reasoning, problem-solving, and language subserve pre-academic skill development. For instance, verbal reasoning is predictive of emerging math and reading competence (Durand et al., 2005), and children’s vocabulary links positive parenting behaviours to later pre-mathematic skills (Wade et al., 2018). Taken together, positive parenting interventions may indirectly affect executive functions and pre-academics through language and mental abilities.

Overall, the current findings illustrate the importance of assessing similarities and differences across outcomes of early development to better understand differential processes of development and intervention effectiveness.

### Causal Pathways

This meta-analysis demonstrates that positive parenting interventions improve the development of children’s mental abilities and language. The use of RCTs allows us to conclude that this relationship is causal. Despite this, there is continued need for further study of mechanism for amplification of theory and clinical application. Though we had intended to examine mechanistic explanations vis a vis positive parenting through pooled mediation analyses (see Prime et al., 2021), this was not feasible. Future research would benefit from assessing indirect effects. For instance, it is unclear which specific elements of positive parenting programs are most influential in improving childhood cognition. Moreover, unmeasured familial processes may be operating (e.g., improvements to parental linguistic input, family routines, parental well-being and/or self-efficacy; Baudry et al., 2015). This is especially a risk for some positive parenting interventions that have minor add-on components.

Relatedly, there are indirect effects that may operate through child self-regulation and/or behavioural problems. Specifically, positive parenting interventions are commonly designed to alter parenting behaviour as a mechanism for addressing children’s behaviour problems (DeGarmo et al., 2004; Dishion et al., 2008; Gardner et al., 2006). Positive parenting programs may directly improve both self-regulation and cognition (via positive parenting), and/or benefits to one child domain may confer benefits to the other. Early parenting programs have a stronger effect on cognition and language as compared to socioemotional, behavioural, and attachment outcomes (Jeong et al., 2021). Furthermore, cognitive abilities are more strongly predictive of children’s self-regulation and behaviour than the reverse (Patwardhan et al., 2021; Peterson et al., 2013; Wang et al., 2018). For these reasons, it is likely that positive parenting interventions directly impact cognition, leading to subsequent improvements in self-regulation and behaviour. However, there is also evidence for a reciprocal relationship between cognition and behavioural development, including in the case of reading and externalizing behaviour (Trzesniewski et al., 2006), and preschool inattention and academic achievement (Metcalfe et al., 2013). Thus, any benefits to behaviour—resulting from improved parenting or cognitive skills—are likely to have benefits for children’s subsequent learning.

All told, there is a need to assess the processes through which positive parenting interventions impact cognitive development, which will inform theoretical and applied perspectives. These lines of inquiry are best addressed through meta-analytic techniques using path analyses involving parenting, behaviour problems, and cognition.

### Moderation Analyses

Next, we consider moderating effects in the meta-analyses examining mental abilities and language—first substantive and then methodological. Of note, there were several moderators that emerged as near-significant, marked in tables that are not discussed here. However, they may signal important differentiators of intervention effectiveness and warrant further evaluation.

#### Substantive Moderators

In the language meta-analysis, positive parenting interventions yielded stronger effect sizes with younger, as compared to older, children. Despite the highly cited idea that ‘earlier is better’ (Heckman, 2008), timing effects in parenting-developmental research have been mixed (Gardner et al., 2019a; Jeong et al., 2021; Sanders et al., 2014). By including infants, toddlers, and preschoolers in the current review, we were able to examine this question using a wide age range in early childhood, which diverges from previous syntheses including only 0–3 years old (Jeong et al., 2021), or children ages 2 years or older (Gardner et al., 2019a, 2019b). The current review provides evidence that, in the case of language development following positive parenting interventions, earlier may be better. That is, intervention effectiveness was reduced for samples of older children, a finding that was robust to simultaneous modeling of other significant predictors. Findings are consistent with another meta-analysis involving parenting interventions for adolescent parents, wherein there was a trend for those involving younger children to show greater intervention effects on children’s cognitive outcomes (Baudry et al., 2017). In contrast, findings diverge from Jeong et al. (2021) recent review. It is enticing to use the two reviews as complementary to one another; there may not be age effects in the first few years of life (ages 0–3 years as in Jeong’s review), with clear distinctions only emerging when comparing infancy to preschool years (as in the current review). Such interpretations must be made with caution, given the differences in inclusion criteria related to the nature of parenting interventions. In the current review, greater change observed in relatively younger children may be due to the rapid development of language skills in the first few years of life, and associated neuroplasticity. That timing effects emerged for language, only, further highlights that such effects may depend on the developmental domain under investigation (Maughan & Barker, 2019).

Interventions were less effective in enhancing child language when implemented with parents with less than or equal to high school education, as compared to those with more than high school education. Positive parenting mediates socioeconomic disparities in early language development (Borairi et al., 2021; Noble et al., 2015). Despite this, the current review does not provide evidence that positive parenting interventions, in isolation, are effective for parents with less than or equal to a high school education. It may be that early language disparities are best supported by programs that target both parental responsiveness and home-literacy/learning activities (Cates et al., 2018; Roby et al., 2021). Alternatively, existing programs may need to be tailored to the sociocultural values, goals, and needs of individual families through a collaborative delivery model to enhance uptake and effectiveness (Gardner et al., 2019a, 2019b; Lunkenheimer et al., 2008)*.* Notably, when modelled simultaneously with other significant moderators, parent education was not a robust moderator. Studies targeting parents with lower levels of formal education may also be initiated later in development (i.e., confounded by the stronger predictor of child age at baseline). Alternatively, parental education may be a true moderator with a small effect that is not robust to a loss in degrees of freedom. In any case, moderation should be interpreted with caution as it reflects associations between studies rather than causal processes.

Notably, only 11 studies (18%) reported father involvement in interventions, a minority proportion consistent with Jeong et al. (2021) recent review. Despite this, father involvement emerged as a significant moderator in the language meta-analysis, wherein studies with some level of father involvement had smaller effect sizes than those without reported father involvement. This finding should be interpreted with caution, given there were few studies in the language meta-analysis that included fathers (*k* = 7) and because the *level* of father involvement could not be ascertained well, based on the reporting in studies. Furthermore, this pattern was not robust to the inclusion of other predictors (child age at baseline). Regardless, further investigation is warranted to assess how father involvement influences intervention effectiveness, given naturalistic evidence linking paternal sensitivity and children’s cognition, learning, and socioemotional adjustment (Rodrigues et al., 2021). Tailoring of interventions may be needed to account for differences amongst mothers and fathers, and to work with interparental couples and children (i.e., triads) rather than focusing on only one parent–child dyad (Nunes et al., 2020).

Overall, there was a dearth of certain high-risk populations, including samples of adolescent parents (*k* = 3) and parents with mental health difficulties (*k* = 10). Given each of these contextual risk factors has been linked to developmental difficulties in children, in part through parenting behaviours (Ahun & Côté, 2019; Firk et al., 2018; Liu et al., 2017), a future endeavour will be to examine the extent to which positive parenting programs can buffer against these contextual risk factors.

#### Methodological Characteristics

For the mental abilities meta-analysis, stronger intervention effects were observed when using standardized direct assessments of child mental abilities, as compared to parent-reported outcome measures. Significant moderation by measurement approach is not uncommon in developmental research, though the pattern of findings has not been consistent. That is, some studies find stronger associations between a predictor/treatment and outcome when observations/direct assessments (rather than parent-report) are used (Madigan et al., 2013), and in other studies the opposite pattern has emerged (Andrews et al., 2021; Nowak & Heinrichs, 2008). Informant discrepancies are likely to inform researchers about meaningful differences across tasks, situations, or contexts, rather than simply reflecting measurement error (De Los Reyes, 2011; De Los Reyes et al., 2009; Kerr et al., 2007). Thus, multi-informant approaches are likely the best approach to comprehensively evaluating intervention effects across settings.

Importantly, outcome measurement approach was reduced to non-significance when modelled simultaneously with risk of bias. Specifically, in the current review, studies with a higher risk of bias yielded stronger intervention effects. This is in line with seminal work demonstrating that low-quality RCTs are associated with increased estimates of benefits of intervention (Moher et al., 1998). This can be said of previous reviews of parenting interventions, as well (Nowak & Heinrichs, 2008; Sanders et al., 2014). This is a critical finding, and divergent from the Jeong (2021) review, which did not identify risk of bias as a significant moderator of effect sizes. This raises significant concerns about the robustness of effect sizes reported for positive parenting interventions in relation to early childhood cognition, given several challenges reported in primary studies based on our risk of bias assessment. Similar to Jeong et al., (2021), the current review identified a crucial need for greater study transparency via pre-registration of hypotheses, outcome assessments, and data analytic plans. Additional issues included poor reporting of a priori power analyses and masking status of data analysts, an important element of psychosocial RCTs. However, strengths identified in the risk of bias assessments (e.g., low risks of selection and detection biases), as well as minimal evidence of publication bias, provide partial support to the reliability of the meta-analysis findings. Furthermore, a correlation analysis showed that more recent studies are associated with a lower risk of bias (*r* = 0.41, *p* < 0.001). Thus, studies are increasingly addressing risk of bias in their study designs and execution. There is no available tool specifically tailored to psychosocial RCTs such as those included in the current meta-analysis. Future development of a refined tool is necessary for more robust risk of bias assessments.

### Limitations

In addition to limitations of primary studies available to the current review, highlighted above, there are limitations due to specific review protocol decisions. First, we only included RCTs, and not non-randomised or single-arm designs. The advantage of this decision was enhanced internal validity, as our primary question related to causal processes involved in positive parenting and early cognition. However, this decision comes with disadvantages, too, including loss of ecological validity (e.g., commonplace settings, representative clinicians), and inclusion of participants who are willing and/or able to participate in random assignment. Future reviews can shed light on systematic differences in the current line of inquiry in randomised versus non-randomised designs.

Second, our review did not include studies when positive parenting interventions were bolstered by other significant program components. Again, this may not reflect the reality of community programming, which integrates several intervention targets with the aim of addressing early disparities in cognitive development. The benefit of this decision is that it uses a single-focused approach; that is, we have identified several circumstances wherein targeting positive parenting, only, is sufficient for improving children’s early mental abilities and language.

Finally, primary studies included in the review were limited to English. Guidelines from organizations such as Cochrane and the Campbell Collaboration do not recommend excluding RCTs reported in languages other than English. However, there is little evidence for increased bias among reviews excluding non-English records in terms of effect estimates and conclusions of systematic reviews (Dobrescu et al., 2021; Moher et al., 2003; Morrison et al., 2012). In any case, this is considered a limitation of the current review.

## Conclusion

Early cognition is an important marker for readiness at school entry, relatively stable across development, and predictive of several adult outcomes in critical domains of education, occupation, health, and well-being. In the current review, positive parenting interventions were effective in promoting positive change in the areas of mental abilities and language. Though effect sizes were smaller and nonsignificant for executive functioning or pre-academics, additional primary studies are required to obtain more precise estimates and examine potential moderators of effectiveness. The current review is the first to isolate positive parenting interventions as an effective approach for enhancing early mental abilities and language based on a synthesis of the extant literature, further underpinning the critical role of parenting interventions for promoting early childhood development.

## Supplementary Information

Below is the link to the electronic supplementary material.

Supplementary file1 (PDF 34 kb)

## Data Availability

The systematic review and meta-analysis was pre-registered at CRD42020222143. PRISMA-P checklist was used to prepare the protocol (Prime et al., 2021) and for reporting in the final report. All data and research materials (screening, full-text assessment, and data extraction manuals) will be available at APA’s repository on the open science framework (OSF) [see Supplemental Materials]. Data were modeled using Comprehensive Meta-Analysis Version 3 and, thus, code/syntax is not applicable.

## Footnotes

1 Primary reasons for exclusion included interventions that provided content directly teaching children skills or focused solely on enhancing parental language input (e.g., shared book reading or literacy interventions) and/or those with significant program components unrelated to positive parenting (e.g., maternal well-being, child health and safety). Minor additional program components were admissible if the primary focus for all participants in the treatment group was positive parenting.

2 This is only a cursory categorization. Though efforts were made to extract measures of risk using a continuous scale (e.g., number of years of education, % of families with low income), primary studies typically did not report this information. Thus, cut-offs were selected based on commonly available information.

3 There were 12 full-texts that could not be located through traditional methods. We attempted to contact authors to request full-texts, and we were successful in obtaining three.

4 We contacted the authors of 61 studies to request additional information such as: means/SD, pre-post correlations or study characteristics. We were successful in obtaining requested information from the authors of 29 studies (the remaining studies had no response (*k* = 18) or decline/unavailable data (*k* = 14).
