# Supplementary material for: Positive Parenting and Early Childhood Cognition: A Systematic Review and Meta-Analysis of Randomized Controlled Trials
Source: Clin Child Fam Psychol Rev. 2023 Feb 2;26(2):362–400. doi: 10.1007/s10567-022-00423-2 (PMC10123053; doi:10.1007/s10567-022-00423-2)
Supplement: Supplementary file 1 — Supplementary file1 (PDF 34 kb) [file 10567_2022_423_MOESM1_ESM.pdf]

Table 1

PsycINFO (OVID) Search Strategy

1. (pre-k* or prek* or infan* or preschool* or toddler* or baby* or babies* or kindergar* or (child* adj6 (young or early))).mp.
2. Family Intervention/
3. Parent Training/
4. Intervention/
5. Early Intervention/
6. ((parent* or mother* or father* or maternal* or paternal* or caregiver*) adj6 (random* or train* or program* or preven* or therap* or treat* or coach* or interve*)).mp.
7. 2 or 3 or 4 or 5 or 6
8. Parenting Skills/
9. Parenting/
10. Mother Child Relations/
11. Parental Characteristics/
12. exp Parents/
13. Parent Child Relations/
14. ((parent* or mother* or father* or maternal* or paternal* or caregiv*) adj6 (proactiv* or contingen* or synchron* or responsiv* or sensitiv* or positiv* or warm* or affect* or support* or involve*)).mp.
15. 8 or 9 or 10 or 11 or 12 or 13 or 14
16. Academic Achievement/
17. School Readiness/
18. exp Childhood Development/
19. exp Cognitive Ability/
20. exp Cognitive Development/
21. (Child* adj6 (develop* or language or linguis* or word* or vocab* or communicat* or execut* or inhibit* or effortful* or cognit* or intellect* or attention* or academic* or numera* or literacy* or read* or spell* or competenc*)).mp.
22. (infan* adj6 (develop* or language or linguis* or word* or vocab* or communicat* or execut* or inhibit* or effortful* or cognit* or intellect* or attention* or academic* or numera* or literacy* or read* or spell* or competenc*)).mp.
23. 16 or 17 or 18 or 19 or 20 or 21 or 22
24. 1 and 7 and 15 and 23
25. limit 24 to (abstract collection or chapter or journal article or publication information)

Note: Date of last search: September 21, 2020; Number of hits: 5560
Date of last search: October 16, 2021; Number of hits: 5970

Table 2

MEDLINE (OVID) Search Strategy

1. exp Infant/
2. exp Child/
3. (pre-k* or prek* or infan* or preschool* or toddler* or baby* or babies* or kindergar* or (child* adj3 (young or early))).mp.
4. 1 or 2 or 3
5. Early Intervention, education/
6. Randomized controlled trial/
7. Treatment Outcome/
8. Psychotherapy/
9. ((parent* or mother* or father* or maternal* or paternal* or caregiver*) adj3 (random* or train* or program* or preven* or therap* or treat* or coach* or interve*)).mp.
10. 5 or 6 or 7 or 8 or 9
11. Parenting/
12. Parents/
13. exp Parent-child relations/
14. Maternal behavior/
15. Paternal behavior/
16. ((parent* or mother* or father* or maternal* or paternal* or caregiv*) adj3 (proactiv* or contingen* or synchron* or responsiv* or sensitiv* or positiv* or warm* or affect* or support* or involve*)).mp.
17. 11 or 13 or 14 or 15 or 16
18. exp Child Development/
19. Cognition/
20. Executive Function/
21. Learning/
22. (Child* adj3 (develop* or language or linguis* or word* or vocab* or communicat* or execut* or inhibit* or effortful* or cognit* or intellect* or attention* or academic* or numera* or literacy* or read* or spell* or competenc*)).mp.
23. (infan* adj3 (develop* or language or linguis* or word* or vocab* or communicat* or execut* or inhibit* or effortful* or cognit* or intellect* or attention* or academic* or numera* or literacy* or read* or spell* or competenc*)).mp.
24. 18 or 19 or 20 or 21 or 22 or 23
25. 4 and 10 and 17 and 24
26. limit 25 to journal article

Note: Date of first search: September 23, 2020; Number of hits: 2628
Date of last search: October 16, 2021; Number of hits: 2932

Table 3

ERIC (ProQuest) Search Strategy

MAINSUBJECT.EXACT.EXPLODE("Children") AND MAINSUBJECT.EXACT("Psychotherapy") OR MAINSUBJECT.EXACT("Early Intervention") OR MAINSUBJECT.EXACT("Randomized Controlled Trials") OR MAINSUBJECT.EXACT("Intervention") OR MAINSUBJECT.EXACT("OUTCOMES OF TREATMENT") OR noft(((parent* OR mother* OR father* OR maternal* OR paternal* OR caregiver*) NEAR/3 (random* OR train* OR program* OR preven* OR therap* OR treat* OR coach* OR interve*))) AND (MAINSUBJECT.EXACT("Parenting Styles") OR MAINSUBJECT.EXACT("Parenting Skills") OR MAINSUBJECT.EXACT("Child Rearing") OR MAINSUBJECT.EXACT("Parent Child Relationship")) OR noft(((parent* OR mother* OR father* OR maternal* OR paternal* OR caregiv*) NEAR/3 (proactiv* OR contingen* OR synchron* OR responsiv* OR sensitiv* OR positiv* OR warm* OR affect* OR support* OR involve*))) AND (MAINSUBJECT.EXACT("Learning") OR MAINSUBJECT.EXACT("Executive Function") OR MAINSUBJECT.EXACT("Cognitive Processes") OR MAINSUBJECT.EXACT.EXPLODE("Child Development") OR MAINSUBJECT.EXACT("School Readiness") OR MAINSUBJECT.EXACT("Academic Achievement") OR MAINSUBJECT.EXACT.EXPLODE("Cognitive Ability") OR MAINSUBJECT.EXACT.EXPLODE("Cognitive Development")) OR (noft(Child* NEAR/3 (develop* OR language OR linguis* OR word* OR vocab* OR communicat* OR execut* OR inhibit* OR effortful* OR cognit* OR intellect* OR attention* OR academic* OR numera* OR literacy* OR read* OR spell* OR competenc*)) OR noft(infan* NEAR/3 (develop* OR language OR linguis* OR word* OR vocab* OR communicat* OR execut* OR inhibit* OR effortful* OR cognit* OR intellect* OR attention* OR academic* OR numera* OR literacy* OR read* OR spell* OR competenc*))) AND stype.exact("Scholarly Journals" OR "Reports" OR "Books" OR "Speeches & Presentations" OR "Conference Papers & Proceedings"

Note: Date of last search: September 23, 2020; Number of hits: 1710
Date of last search: October 16, 2021; Number of hits: 1903

Table 4

Dissertations & Theses Global (ProQuest)
noft(((parent* OR mother* OR father* OR maternal* OR paternal* OR caregiver* OR famil*) NEAR/3 (random* OR train* OR program* OR preven* OR therap* OR treat* OR coach* OR interve*))) AND noft(((parent* OR mother* OR father* OR maternal* OR paternal* OR caregiv*) NEAR/3 (relations* OR behav* OR proactiv* OR contingen* OR synchron* OR responsiv* OR sensitiv* OR positiv* OR warm* OR affect* OR support* OR involve*))) AND noft(Child* NEAR/3 (develop* OR language OR linguis* OR word* OR vocab* OR communicat* OR execut* OR inhibit* OR effortful* OR cognit* OR intellect* OR attention* OR academic* OR numera* OR literacy* OR read* OR spell* OR competenc* OR learn* OR IQ)) OR noft(infan* NEAR/3 (develop* OR language OR linguis* OR word* OR vocab* OR communicat* OR execut* OR inhibit* OR effortful* OR cognit* OR intellect* OR attention* OR academic* OR numera* OR literacy* OR read* OR spell* OR competenc* OR learn* OR IQ)) AND la.exact("ENG")

Note: Date of last search: September 23, 2020; Number of hits: 2277
Date of last search: October 16, 2021; Number of hits: 2424
